# Region-specific activation in the accumbens nucleus by itch with modified scratch efficacy in mice – a model-free multivariate analysis

**DOI:** 10.1186/s13041-024-01101-w

**Published:** 2024-05-23

**Authors:** Sanae Inokuchi-Sakata, Ryo Narita, Yukari Takahashi, Yozo Ishiuji, Akihiko Asahina, Fusao Kato

**Affiliations:** 1 Department of Dermatology, The Jikei University School of Medicine, Minato-Ku, Tokyo, 105-8461 Japan; 2 Department of Neuroscience, The Jikei University School of Medicine, Minato-ku, Tokyo 105-8461 Japan; 3 Center for Neuroscience of Pain, The Jikei University School of Medicine, Minato-Ku, Tokyo, 105-8461 Japan

**Keywords:** Hierarchical cluster analysis, Dopamine receptors, Multiplex fluorescence in situ hybridization, RNAscope, Histamine, Nail clipping, c-fos

## Abstract

Itch is a protective/defensive function with divalent motivational drives. Itch itself elicits an unpleasant experience, which triggers the urge to scratch, relieving the itchiness. Still, it can also result in dissatisfaction when the scratch is too intense and painful or unsatisfactory due to insufficient scratch effect. Therefore, it is likely that the balance between the unpleasantness/pleasure and satisfaction/unsatisfaction associated with itch sensation and scratching behavior is determined by complex brain mechanisms. The physiological/pathological mechanisms underlying this balance remain largely elusive. To address this issue, we targeted the "reward center" of the brain, the nucleus accumbens (NAc), in which itch-responsive neurons have been found in rodents. We examined how neurons in the NAc are activated or suppressed during histamine-induced scratching behaviors in mice. The mice received an intradermal injection of histamine or saline at the neck, and the scratching number was analyzed by recording the movement of the bilateral hind limbs for about 45 min after injection. To experimentally manipulate the scratch efficacy in these histamine models, we compared histamine's behavioral and neuronal effects between mice with intact and clipped nails on the hind paws. As expected, the clipping of the hind limb nail increased the number of scratches after the histamine injection. In the brains of mice exhibiting scratching behaviors, we analyzed the expression of the c-fos gene (Fos) as a readout of an immediate activation of neurons during itch/scratch and dopamine receptors (Drd1 and Drd2) using multiplex single-molecule fluorescence in situ hybridization (RNAscope) in the NAc and surrounding structures. We performed a model-free analysis of gene expression in geometrically divided NAc subregions without assuming the conventional core–shell divisions. The results indicated that even within the NAc, multiple subregions responded differentially to various itch/scratch conditions. We also found different clusters with neurons showing similar or opposite changes in Fos expression and the correlation between scratch number and Fos expression in different itch/scratch conditions. These regional differences and clusters would provide a basis for the complex role of the NAc and surrounding structures in encoding the outcomes of scratching behavior and itchy sensations.

**Supplementary Information:**

The online version contains supplementary material available at 10.1186/s13041-024-01101-w.

## Introduction

Itch, an unpleasant sensation on the skin that provokes the desire to scratch [1], serves an essential protective function by detecting harmful conditions, promoting an unpleasant affection to sound an alarm, and facilitating their removal through scratching [2, 3]. This function is realized through multidimensional elements involving sensory perception, unpleasant affection/emotion, and the resulting urge/motivation to scratch. Most importantly, scratching an itch may be intensely pleasurable and rewarding, which forms a strong driving force for the urge to scratch. Consequently, this intense desire to scratch provides a basis for the itch-scratch cycle, which may become addictive and vicious, particularly with chronic itch [4], such as in patients with atopic dermatitis [3]. To stop the vicious itch-scratch cycle in chronic itch, further studies are needed to elucidate the brain mechanisms underlying the urge to scratch caused by an itchy sensation and to clarify how scratching relieves this urge and promotes pleasure and satisfaction.

Among the various mechanisms responsible for desire and satisfaction, the accumbens nucleus (NAc), located bilaterally in the midbrain, plays a pivotal role in the dopamine-regulated behaviors associated with rewards [5]. The NAc is involved in euphonia and drug dependence [6], and dopaminergic inputs from the ventral tegmental area (VTA) to the NAc regulate pleasure-oriented behaviors [7]. Neurons in the NAc are activated in response to various stimuli and environments, providing rewards in humans and animals [8, 9]. A recent study revealed that dopaminergic projections from the VTA to the lateral shell of the NAc regulate scratching behaviors [10]. In addition, functional magnetic resonance imaging studies on humans showed that scratching with pleasure was accompanied by the activation of midbrain regions, including the VTA, striatum, and NAc [3, 11]. However, the mechanisms underlying the transformation of itch-associated unpleasantness to pleasantness by scratching remain largely elusive.

In addition, the intra-nucleus organization of the NAc in relation to its behavioral roles has never been clearly demonstrated. Conventionally, the NAc has been classified into dichotomic regions, such as the core-and-shell and lateral-and-medial, according to the rough location relative to the olfactory limb of the anterior commissure (aco) in coronal sections [12]. Studies using local microinjection of drugs and electrophysiological recordings have revealed regional differences of the NAc, particularly in the lateral shell, associated with itch and scratch behaviors [10, 13, 14]. However, the rough spatial resolutions of the techniques and limited experimental access to various small regions within and around the NAc hindered detailed analysis of the function of each region in itch and scratch behaviors. In addition, only limited information has been provided about the rostrocaudal differences in terms of itch and scratch behaviors [14].

Therefore, the present study attempted to identify the subregions and neighboring structures of the NAc activated or inactivated during scratching behaviors associated with the scratching-induced outcomes (i.e., scratch efficacy) and itch relief [15]. To achieve this goal, we observed scratching behaviors in mice after an intradermal injection of histamine or saline at the neck and analyzed the expression of the c-Fos gene (*Fos*), an immediate-early gene expressed in response to neuronal excitation, together with that of dopamine receptors (Drd1 and Drd2), which play essential roles in the regulation of NAc activity [16, 17] using multiplex single-molecule fluorescence in situ hybridization (FISH). We also compared the effects of histamine between mice with intact and clipped nails on the bilateral hind paws to manipulate the scratch efficacy experimentally [18, 19]. We employed a "model-free" approach, i.e., without postulating the conventional anatomical classification for itch/scratch, such as the core and shell, which has only roughly been defined without any strict morphofunctional distinctions [12], to statistically analyze differences in expression levels between experimental conditions using a hierarchical cluster analysis of populations of Fos-expressing cells in each non-subjectively defined subregion. The spatially clustered activation patterns revealed with the present approach did not necessarily correspond with the conventional "core-and-shell" classification [20]. This approach also revealed that the borders between the NAc and surrounding structures cannot be clearly defined. To express this condition, we used the term "extended NAc" (eNAc) hereinafter to describe the results obtained. We demonstrated that multiple subregions formed functionally independent clusters in response to various itch/scratch conditions, providing a basis for the complex role of the eNAc in scratching behavior and itchy sensations.

## Results

### The histamine injection at the nape increased scratching behaviors in a manner that was dependent on the hindlimb nail status

**Fig. 1:**
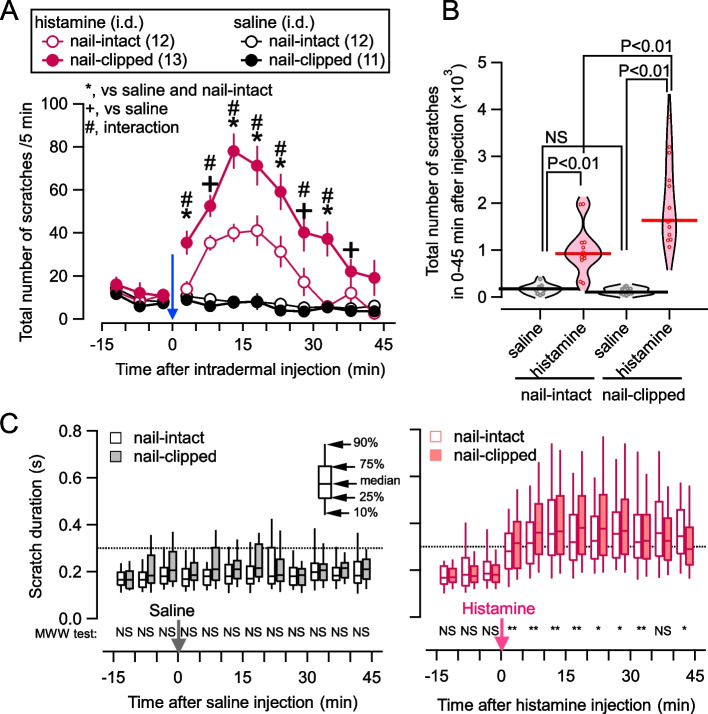
Effects of an intradermal injection of histamine and nail clipping on scratching behavior. **A** Time course of the total number of scratches pooled observed in every five minutes after an intradermal injection of histamine/saline at time 0 (blue arrow). Red and black markers indicate the histamine- and saline-injected groups, respectively. Open and filled circles indicate the nail-intact and nail-clipped groups. The numbers in parentheses in the upper box are the number of mice in each group. Statistical comparisons were performed with a two-way ANOVA: *, *P* < 0.05 for histamine/saline injection and nail-intact/clipped; + , *P* < 0.05 only for histamine/saline injection; #, *P* < 0.05 interaction between histamine/saline and nail-clipped/intact. Mean ± SEM. **B** Violin plots of the total number of scratches for 45 min after the injection of histamine/saline in all mice used for Fig. 1A. Circles indicate the number of scratches in each mouse. Horizontal bars indicate the average in each group. Statistical comparisons were performed with a one-way ANOVA (post hoc analysis with Bonferroni). NS: not significant. **C** The box-and-whisker plots of the scratch duration of each bout after the injection of saline (left) and histamine (right). Open and filled boxes indicate the scratch duration pooled by five minutes in nail-intact and nail-clipped groups, respectively. The boxes and whiskers represent distributions as illustrated in the inset in the left panel. Dotted lines are drawn at 0.3 s, i.e., the 10%-upper confidential limit of the duration before the histamine/saline injection (-15 ~ 0 min). Statistical comparisons were performed with a Mann–Whitney-Wilcoxon test. * *P* < 0.05, ** *P* < 0.01, NS: not significant. The box-and-whisker plots are constructed using the full set of detected scratch bouts, of which the mean values over 11–13 mice are shown in Fig. 1A

We measured scratching behaviors in all mice belonging to either of four groups: with or without nail-clipping after intradermal histamine or saline injection for 45 min. The details of the preparation and measurements are described in Fig. S1 and Methods. Mice in all four cohorts always presented “spontaneous” scratching behaviors (Fig. 1A; -15 – 0 min) before injections. This spontaneous scratching did not significantly differ between nail-clipped and nail-intact mice (Fig. 1A, filled and open circles). As expected, the intradermal injection of histamine significantly increased the number of scratches both in nail-clipped and nail-intact groups, with a significantly larger number in nail-clipped mice than in nail-intact mice (Fig. 1A and B). To examine whether the scratch behavior was modified not only quantitatively but also qualitatively by nail-clipping, we analyzed the duration of each scratch bout. The increase in the number of scratches after histamine injection was accompanied by an increase in the scratch duration with a shift of the distribution toward longer ones (Fig. 1C right) and this shift in distribution was significantly larger in nail-clipped mice (Fig. 1C right). This might have resulted from the compensatory increase in scratch demand due to the lowered efficacy of scratches with clipped nails. Apparent damage to the skin surface was not detected by the observation by experienced dermatologists (S.I.S and Y.I.) in a blinded manner after the 60-min post-histamine or post-saline observation regardless of whether the nail was clipped or intact, as previously reported in the model with a single injection of histamine injection model [21]. Thus, these four conditions of scratch behavior observations, i.e., the histamine vs. saline injection with clipped vs. intact nails, would represent distinct brain states associated with itch sensation, scratches, and their outcomes. We analyzed the activities of NAc, using the c-Fos expression as a proxy, together with dopamine receptor expressions, by comparing these four conditions below.

### Expression of Fos and dopamine receptor mRNA in the NAc and surrounding structures in mice after scratching behavior

**Fig. 2:**
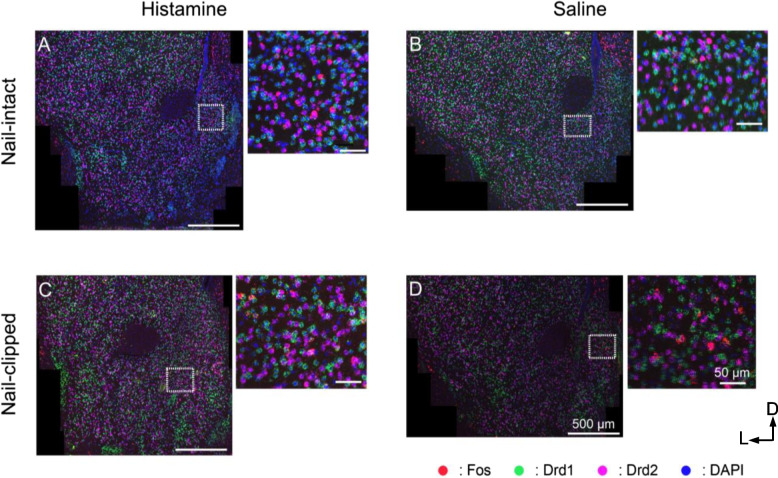
Distribution of Fos, Drd1, and Drd2 RNAscope signals in the extended NAc (eNAc). Representative multiplex in situ hybridization images of the eNAc in histamine-injected nail-intact (**A**), saline-injected nail-intact (**B**), histamine-injected nail-clipped (**C**), and saline-injected nail-clipped (**D**) mice. All panels are original microscope images with four channels: Fos (red), Drd1 (green), Drd2 (magenta), and DAPI (blue). A magnified image of the dashed rectangle on the left image is shown on the right side. Scale bars indicate 500 µm (left) and 50 µm (right). The top and left in all images are dorsal and lateral, respectively

**Fig. 3:**
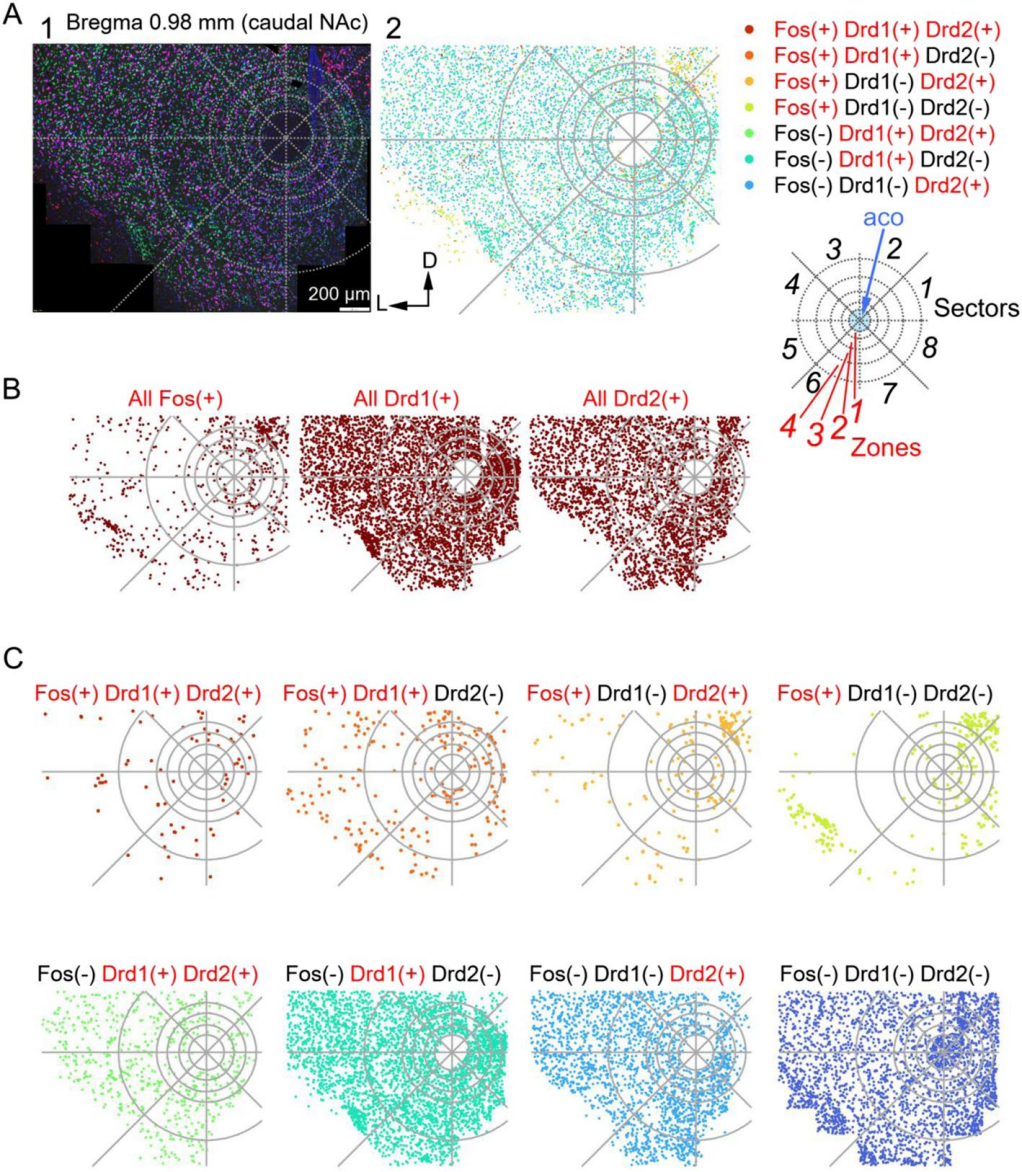
Representative plots for molecular expression in the eNAc in nail-intact saline-injected mice. **A** An example of an original microscopic image using RNAscope (A1) and the distribution of Drd1-, Drd2-, and Fos-expressing cells (A2) in eNAc. The eNAc was divided into 32 zone-sectors (zones 1–4 and sectors 1–8) with dashed circles and lines, as depicted on the right side (see METHODS and Supplementary figures). One dot represents one cell. The colors of dots in A2 indicate the combination of Drd1, Drd2, and Fos mRNA expression patterns as indicated at the right top. aco, anterior commissure. **B** The distribution of positive cells for Fos (left), Drd1 (middle), and Drd2 (right) mRNA in the slice in Fig. 3A. **C** The distribution of cells showing the same mRNA expression patterns in the slice in Fig. 3A. The combination of the molecules expressed is described at the top of each plot. The rightmost lower panel indicates the distribution of negative cells for Drd1, Drd2, and Fos

To clarify the roles of the subregions of the so-called "pleasantness" center of the brain, namely, the NAc and surrounding structures (hereinafter called the eNAc), we removed the brains from mice in the four cohorts immediately after the 45-min measurement of scratching behaviors and analyzed the expression of Fos together with that of the Drd1 and Drd2 receptors using multiplex in situ hybridization (RNAscope; Fig. 2). Instead of using a conventional atlas, we applied the following three coordinate dimensions to specify locations in and around the eNAc: 1) *Zones*: four concentric zones bounded by five circles ("zones") with the center of the aco [20]. The aco is a compact round-shaped structure in coronal sections, rich in rostrocaudal passing fibers devoid of a neuron soma. These circular-shaped zones were numbered 1 to 4 from the center to the periphery (Fig. 3A and Fig. S3E). 2) *Sectors*: eight "sectors," each with a 45° angle, starting from the sector immediately above the horizontal line and most medial (sector 1) and numbered counter-clockwise to sector 8 (Fig. 3A). 3) *Planes*: three anterior–posterior sections from the rostral, intermediate (hereinafter abbreviated as "intermed"), and caudal eNAc (Fig. S3E).

Figure 3A2, 3B, and 3C show representative examples of RNAscope images of the eNAc in the caudal plane from a nail-intact mouse after the saline injection. As shown in each panel in Fig. 3C, the mRNA expression of Fos, Drd1 and Drd2 together with the signal with 4',6-diamidino-2-phenylindole (DAPI) (Fig. 3A1) were visualized. Based on the expression of each molecule and their co-expression in all eNAc subregions, we calculated the fraction of cells expressing Fos in each region by dividing the number of Fos-expressing cells by the number of cells marked with DAPI in each region.

### The histamine injection significantly affected Fos expression in subregion- and nail condition-dependent manners

**Fig. 4:**
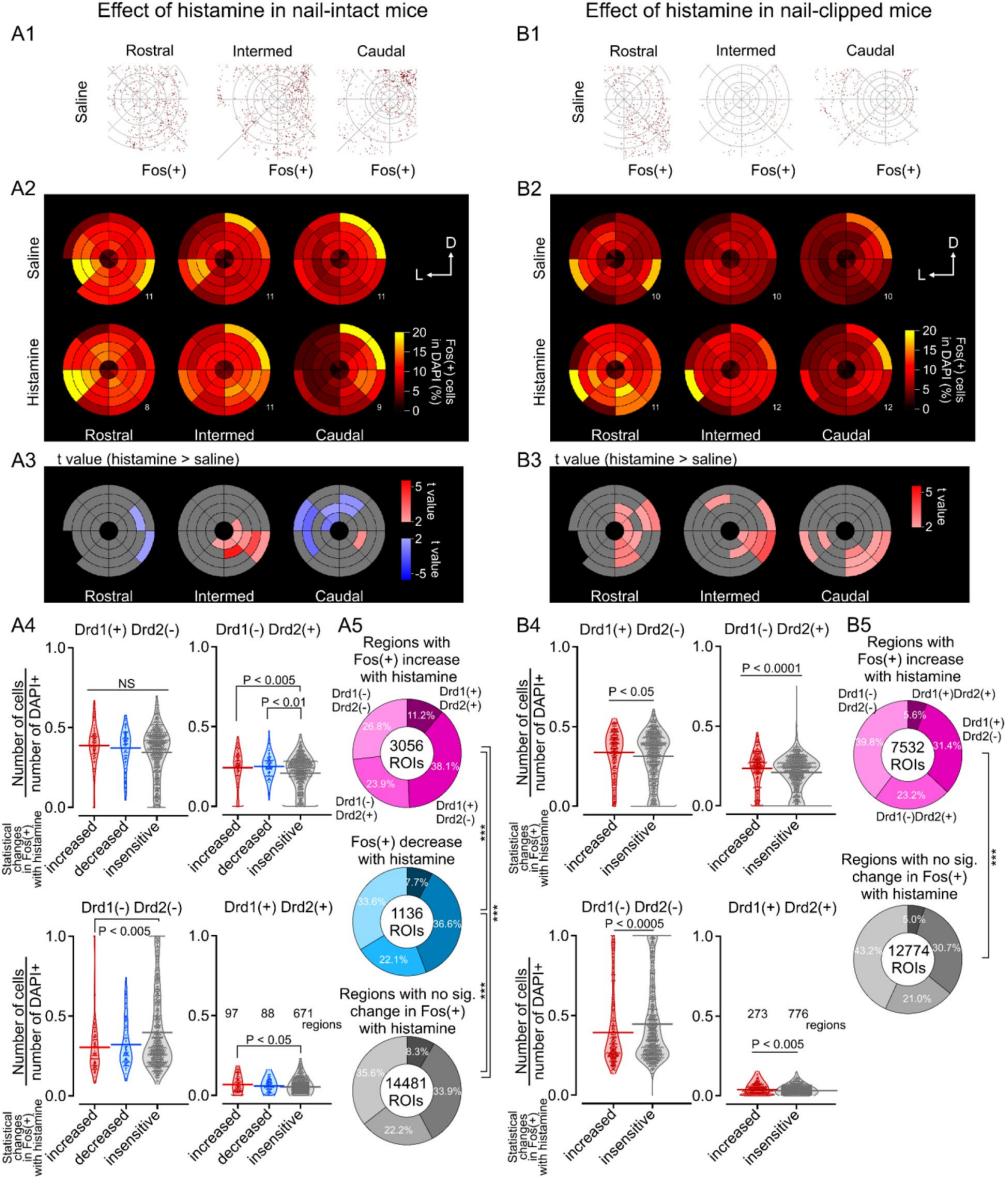
Regional differences in Fos expression in the eNAc induced by the intradermal injection of histamine. Fos expression in nail-intact (**A**) and nail-clipped (**B**) mice in the saline- and histamine-injected groups. A1, B1. Representative Fos( +) cell distribution in the eNAc of a saline-injected mouse. A2, B2. Zone-and-sector plots of Fos expression in the eNAc of saline- (upper) or histamine- (lower) injected mice. Color-coded percentages indicate the mean of the fraction of Fos( +) cells over DAPI( +) cells. Numbers in the right bottom of each zone-and-sector plot indicate the number of cases (one case = 1 slice from a mouse). A3, B3. Zone-and-sector plots indicating the t values of statistical comparisons. Reddish and bluish colors indicate regions with an increase and decrease in Fos expression in histamine-injected mice, respectively (regions of Q < 0.1 after the false discovery rate correction are shown). A4, B4. Comparison of the fractions of dopamine receptor-positive cells in three types of eNAc regions with different responses to histamine. Fractions of cells with each dopamine receptor are plotted as dots and violins (horizontal lines, mean). Regions were classified into "increased", "decreased", and "insensitive" expression in Fos( +) cells after the histamine injection. The numbers of valid regions for the analysis were described in the lower-right graph. The Kruskal–Wallis test with a post hoc analysis with the Bonferroni (Fig. 4A4) or Mann–Whitney U test (Fig. 4B4) was performed. P values indicating significant differences are described. NS, not significantly different. A5, B5. The composition of cells expressing dopamine receptors in Fos( +) cells in "increased", "decreased", and "insensitive", regions. The numbers of Fos( +) cells are described at the center of circles. The percentages of the four patterns of dopamine receptor co-expression in Fos( +) cells were compared between regions with different histamine responses using the chi-square test. ***, *P* < 0.005

Figure 4A1 shows a typical example of the expression map of Fos in the rostral, intermed, and caudal eNAc planes in a saline-injected nail-intact mouse. After counting the numbers of Fos( +) cells and DAPI( +) cells in each region, we calculated the fraction of Fos( +) cells in DAPI( +) cells in each region in mice in each of the four cohorts. Figure 4A2 shows the mean fraction of Fos( +) in DAPI( +) cells in each of the four zones and eight sectors in the three different planes (from left to right) in nail-intact mice intradermally injected with saline (above) and histamine (below). We called these plots "zone-and-sector plots", which were used to indicate the mean values of measures (i.e., the mean fraction of Fos-expressing cells and the coefficient of correlation) for each subregion. Depending on the rostrocaudal level, some regions were not used in the cell count evaluation because of insufficient images in that region (e.g., zone 4-sector 5 in the rostral plane of saline-injected nail-intact mice).

Contrary to our expectations, a high fraction of Fos( +) was observed in eNAc regions in saline-injected mice, suggesting that eNAc neurons were activated even during "housekeeping" states with spontaneous scratching behaviors (Fig. 4A2 above). In histamine-injected mice (Fig. 4A2 below), the degree of increase in Fos(+) expression was region-dependent (expressed in the yellow-red color scale). To examine differences in the Fos( +) cell fraction between saline- and histamine-injected mice, differences in expression levels were statistically compared. Figure 4A3 shows the t values of saline-histamine comparisons (positive t values are shown in a red scale and negative t values in a blue scale, indicating larger and smaller t values in histamine-injected mice than in saline-injected mice, respectively). The medial core regions in the intermed plane showed significantly larger t values (red, positive t values) in histamine-injected mice, while the dorsolateral regions in the caudal plane showed smaller t values (blue, negative t values). Fos expression patterns in the eNAc of nail-clipped mice differed from those in nail-intact mice (Fig. 4B). In contrast to the effects of histamine in nail-intact animals, histamine significantly increased the Fos( +) fraction in all regions with significant changes (Fig. 4B2 and B3), indicating that the lowered outcome of scratching with clipped nails exerted stronger activating effects on eNAc subregions. This effect was prominent in the medial and ventral parts of the NAc regardless of the shell or core (Fig. 4B3). The medial core and shell regions in the rostral, intermed, and caudal planes exhibited significantly larger t values (red, positive t values) in histamine-injected nail-clipped mice. These results indicate that the cellular responses of NAc neurons in the elicited itchiness were dependent on the regions and were not consistent within eNAc subregions. This strongly supports the notion that the role of the NAc in the itchiness-scratch relationship is not consistent throughout NAc subregions, rather it is specific to clustered subregions.

### The composition of two dopamine receptor types in regions with distinct changes in Fos expression by histamine significantly differed

To investigate whether the expression patterns of the two different types of dopamine receptors affected distinct Fos responses in different regions following the histamine injection, we compared the expression type of dopamine receptors in regions with "increased", "decreased", and no significant change (hereinafter called "insensitive") in Fos expression in histamine-injected mice than in saline-injected mice (Fig. 4A4, A5, B4, B5). A total of 856 and 1049 regions in Fig. 4A and B were identified in the "zone-and-sector" analysis of rostral (8 and 11 sections for Fig. 4A and B, respectively), intermed (11 and 12 sections), and caudal slices (9 and 12 sections) from nail-intact (12 mice, Fig. 4A) and -clipped (13 mice, Fig. 4B) histamine-injected mice. These regions were classified into that with a significant increase, decrease, or insensitive in Fos expression according to the statistical results shown in Fig. 4A3 and B3. The percentage of Drd1(-) Drd2(-) cells was lower, while that of Drd1( +) Drd2( +) cells was higher in regions with increased Fos than in regions with insensitive Fos expression in nail-intact mice (Fig. 4A4).

In contrast, in nail-clipped mice, regions with increased Fos expression showed higher percentages of Drd1( +) Drd2(-), Drd1(-) Drd2( +), and Drd1( +) Drd2( +) cells than the insensitive regions (Fig. 4B4), while the percentage of Drd1(-) Drd2(-) cells was significantly lower, suggesting that Fos increased regions are characterized by the expression of D1 and D2 receptors (D1R and D2R, respectively).

The regions analyzed in Fig. 4A4 and B4 contained 18,673 and 20,306 regions of interest (ROIs), respectively, for cells expressing Fos. We classified these Fos-expressing ROIs into four subgroups according to the expression patterns of Drd1 and Drd2 in each of these ROIs. These four subgroups were 1) Drd1( +)Drd2( +), ROIs expressing genes for both D1Rs and D2Rs, 2) Drd1( +)Drd2(-), 3) Drd1(-) Drd2( +), and 4) Drd1(-)Drd2(-), ROIs expressing none of these dopamine receptors, but only Fos. We then compared the ratio of dopamine receptor expression patterns co-expressed with Fos in each region between regions with increased, decreased and insensitive Fos expression to clarify whether these differences in Fos expression were related to different expression patterns of dopamine receptors (Fig. 4A5 and B5). Magenta, blue, and gray pie charts indicate the composition of the expression patterns of dopamine receptors in Fos-increased, -decreased, and -insensitive regions, respectively, in histamine-injected mice. A multiple chi-square comparison indicated that the percentages of the four expression patterns of dopamine receptors significantly differed between the regions with distinct responses to the histamine injection, suggesting that the activation of subregions in the eNAc by histamine reflected differences in the expression patterns of the two dopamine receptors in both nail-intact (Fig. 4A5) and nail-clipped mice (Fig. 4B5).

The expression fraction of distinct types of dopamine receptor mRNAs in the eNAc is shown in Fig. S5. Overall, the expression of Drd1( +) cells was slightly higher than that of Drd2( +) cells throughout the eNAc. Although their number was lower, the co-expression of Drd1( +) Drd2( +) cells was also observed regardless of the regions (Fig. S5B). 

### Lower number of Fos( +) cells in a subset of eNAc regions in nail-clipped mice than in nail-intact mice injected with saline and histamine

**Fig. 5:**
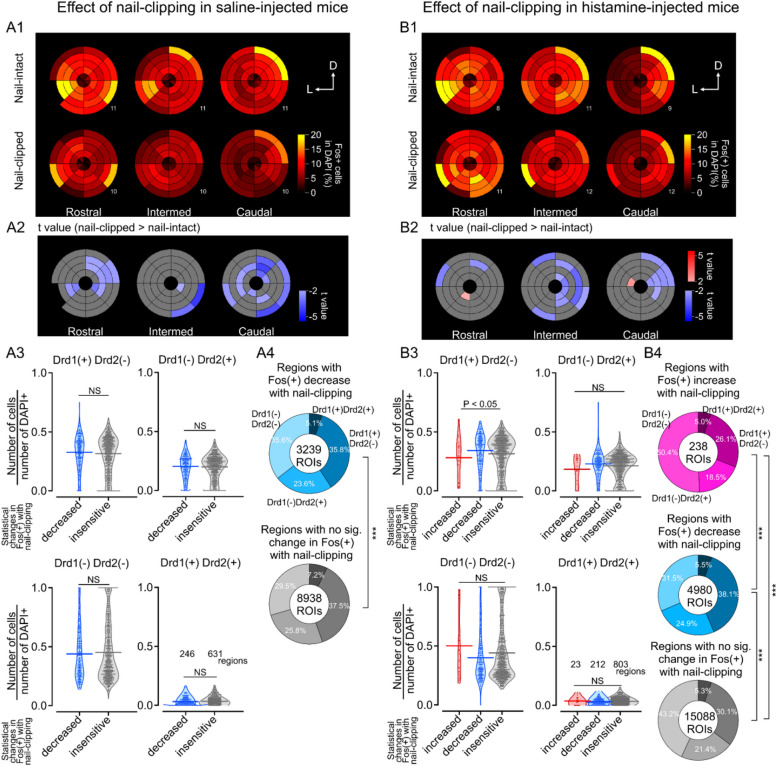
Regional differences in Fos expression in the eNAc induced by nail clipping. Fos expression in saline- (**A**) and histamine- (**B**) injected mice compared between the nail-intact and nail-clipped groups. A1, B1. Zone-and-sector plots of Fos expression in the eNAc of nail-intact (upper) or nail-clipped (lower) mice. Color-coded percentages indicate the mean of the fraction of Fos( +) cells over DAPI( +) cells. Numbers in the right-bottom of each zone-and-sector plot indicate the number of cases (one case = 1 slice from a mouse). A2, B2. Statistical zone-and-sector plots comparing Fos expression in the eNAc between nail-intact and nail-clipped mice. Reddish and bluish regions indicate higher and lower Fos expression in nail-clipped mice, respectively (regions of Q < 0.1 after the false discovery rate correction (Benjamini-Hochberg) colored with the t value). A3, B3. Comparison of the fractions of dopamine receptor-positive cells in three types of eNAc areas with different reactiveness to nail clipping. Fractions of cells with each dopamine receptor in increased, decreased, and insensitive regions in the nail-clipped group were plotted as dots and violins (horizontal lines, the mean). The numbers of valid regions for the analysis are shown in the lower-right graph in Fig. 5A3 and B3. The Mann–Whitney U test. P values are described for comparative pairs with significant differences only. NS, not significantly different. A4, B4. The composition of cells expressing dopamine receptors in the Fos( +) group in different regions with different reactivity to nail clipping in nail-clipped mice. The numbers of Fos( +) cells are described in the center of circles. The percentages of the four patterns of dopamine receptor co-expression in Fos( +) cells were compared between regions with different responses to nail-clipping using the chi-square test. ***, *P* < 0.005

We then examined the effects of nail clipping on Fos expression in mice with spontaneous (with saline) and elicited (with histamine) scratching behaviors (Fig. 5). Nail clipping consistently decreased the fraction of Fos( +) cells depending on the regions examined, particularly in the caudal and dorsomedial rostral eNAc in saline-injected mice (blue-colored regions in the zone-and-sector plots in Fig. 5A2). Decreased Fos expression in nail-clipped mice was also observed in histamine-injected mice, except in two small regions with significantly higher Fos expression near the aco (Fig. 5B2). However the rostro-caudal distribution of regions with a significant decrease in Fos( +) differed in saline- and histamine-injected mice (Fig. 5A2 and B2), suggesting that the effects of nail clipping on neuronal activities were represented in distinct anterior–posterior sites in spontaneous and elicited itchiness. A total of 877 and 1038 regions for Fig. 5A and B were identified in the "zone-and-sector" analysis for rostral (10 and 11 sections for Fig. 5A and B, respectively), intermed (10 and 12 sections), and caudal slices (10 and 12 sections) from nail-clipped mice with the saline-injected (11 mice, Fig. 5A) and histamine-injected (13 mice, Fig. 5B). Despite the significantly lower expression of Fos by nail clipping in saline-injected mice, the types of dopamine receptors expressed across regions did not significantly differ regardless of differences in Fos expression (Fig. 5A3). In contrast, in histamine-injected mice, regions with a significant decrease in Fos expression by nail clipping showed a significantly higher percentage of Drd1( +) Drd2(-) cells. (Fig. 5B3), suggesting a role for D1R-mediated signaling in the effects of nail clipping.

The percentage of Fos-expressing cells with the distinct expression patterns of two dopamine receptor subtypes (Drd1( +) Drd2( +), Drd1( +) Drd2(-), Drd1(-) Drd2( +), and Drd1(-) Drd2(-)) also differed between regions with and without significant Fos expression (Fig 5A4 and B4). Magenta, blue, and gray pie charts show the composition of expression patterns of dopamine receptors in Fos-increased, -decreased, and -insensitive regions, respectively, in nail-clipped mice.

The regions analyzed in Fig. 5A4 and B4 contained 12,177 and 20,306 ROIs, respectively. A multiple chi-square comparison indicated that the percentages of the four expression patterns of dopamine receptors significantly differed between the regions with distinct responses to nail clipping, suggesting that the activation of subregions in the eNAc by nail clipping reflected differences in the expression patterns of the two dopamine receptors in both saline- (Fig. 5A4) and histamine-injected mice (Fig. 5B4). These results indicate that region-dependent differences in nail clipping-associated decrease in Fos expression were related to the type of dopamine receptor expressed in each region.

### eNAc regions were classified into five clusters according to changes in the Fos( +) fraction in response to nail clipping and the histamine injection

**Fig. 6:**
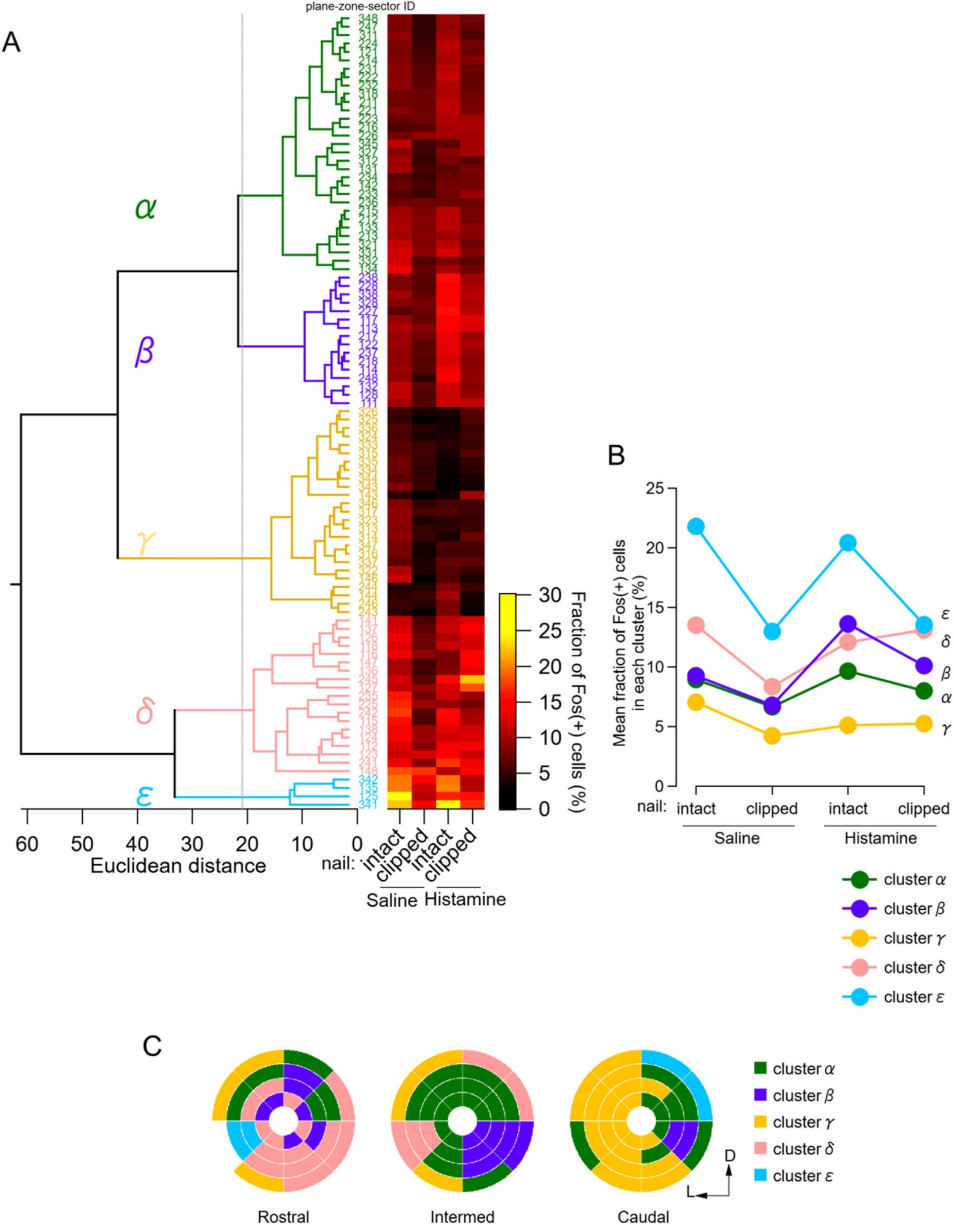
Hierarchical cluster analysis to categorize eNAc regions by Fos expression patterns under different itch/scratch conditions. **A** Left: A dendrogram representing clusters based on the Fos expression data of 95 regions (4 zones × 8 sectors × 3 planes, except for zone 4-sector 5 in the rostral slice). The vertical dashed line indicates the Euclidean distance threshold used to separate the regions into 5 independent clusters. The three-digit numbers to the right of the dendrogram correspond to plane-zone-sector IDs (Table 1). Right: Heat map plot showing the mean fraction of Fos( +) cells in each region under four itch/scratch conditions. The heatmap on the left shows the fraction of Fos( +) cells in each region using the color scale on the right. **B** The mean fraction of Fos( +) cells in each cluster under the different itch/scratch conditions. The numbers below the figure indicate the cluster numbers shown in Fig. 6A. **C** Zone-and-sector plots showing the clusters to which each region belongs. Cluster numbers are shown with a color index on the right

As shown above, each NAc subregion responded in different manners to histamine and saline under the nail-clipped and -intact conditions. The patterns of increases and decreases in Fos expression markedly differed between the regions, were dependent on nail conditions, and were complex. To identify the patterns of regions responding to distinct conditions with commonly shared specific patterns, we performed a hierarchical cluster analysis to categorize the groups showing similar changes in Fos expression (Fig. 6A). We identified five main clusters with different response patterns in the four cohorts of treatments and conditions. Figure 6B shows the average percentage of Fos( +) cells in regions classified to each cluster (the cluster classification of each region is shown in Fig. 6C) in response to each treatment. Their responses are summarized as follows: *Cluster ε* (the mediodorsal caudal NAc, shown in blue in the dendrogram in Fig. 6A, blue filled-circles in Fig. 6B, and blue subregions in Fig. 6C) was characterized by the higher expression of Fos, particularly when the nails were intact, in both the saline- and histamine-injected groups, *Cluster γ* (the lateral caudal NAc, shown in yellow) had consistently low Fos expression regardless of the treatment (low responsiveness), *Cluster α* (the dorsal core intermed NAc, shown in green) exhibited low Fos activity, with markedly lower Fos expression when the nails were clipped (low, but nail-dependent), *Cluster δ* (centered around the ventromedial shell in the rostral NAc, shown in peach) showed lower Fos expression only when the nails were clipped in the saline-injected cohort (high, except for nail-clipped + saline), and *Cluster β* (centered around the ventromedial NAc at the intermed level, mostly overlapping with the conventional medial core, shown in violet) had an interesting pattern; neural activity was enhanced by the histamine injection, but decreased upon nail clipping (nail- and histamine-dependent). In summary, regions with similar responses were more likely to cluster in anatomically proximate areas of the eNAc (Fig. 6C).

**Table 1:** Three-digit ID codes for plane-zone-sector IDs

PZS ID	Plane	Zone	Sector	PZS ID	Plane	Zone	Sector	PZS ID	Plane	Zone	Sector
111	Rostral	1	1	211	Intermed	1	1	311	Caudal	1	1
112			2	212			2	312			2
113			3	213			3	313			3
114			4	214			4	314			4
115			5	215			5	315			5
116			6	216			6	316			6
117			7	217			7	317			7
118			8	218			8	318			8
121		2	1	221		2	1	321		2	1
122			2	222			2	322			2
123			3	223			3	323			3
124			4	224			4	324			4
125			5	225			5	325			5
126			6	226			6	326			6
127			7	227			7	327			7
128			8	228			8	328			8
131		3	1	231		3	1	331		3	1
132			2	232			2	332			2
133			3	233			3	333			3
134			4	234			4	334			4
135			5	235			5	335			5
136			6	236			6	336			6
137			7	237			7	337			7
138			8	238			8	338			8
141		4	1	241		4	1	341		4	1
142			2	242			2	342			2
143			3	243			3	343			3
144			4	244			4	344			4
145			5	245			5	345			5
146			6	246			6	346			6
147			7	247			7	347			7
148			8	248			8	348			8

### The relationship between the number of scratches and Fos expression under each condition was dependent on eNAc regions

**Fig. 7:**
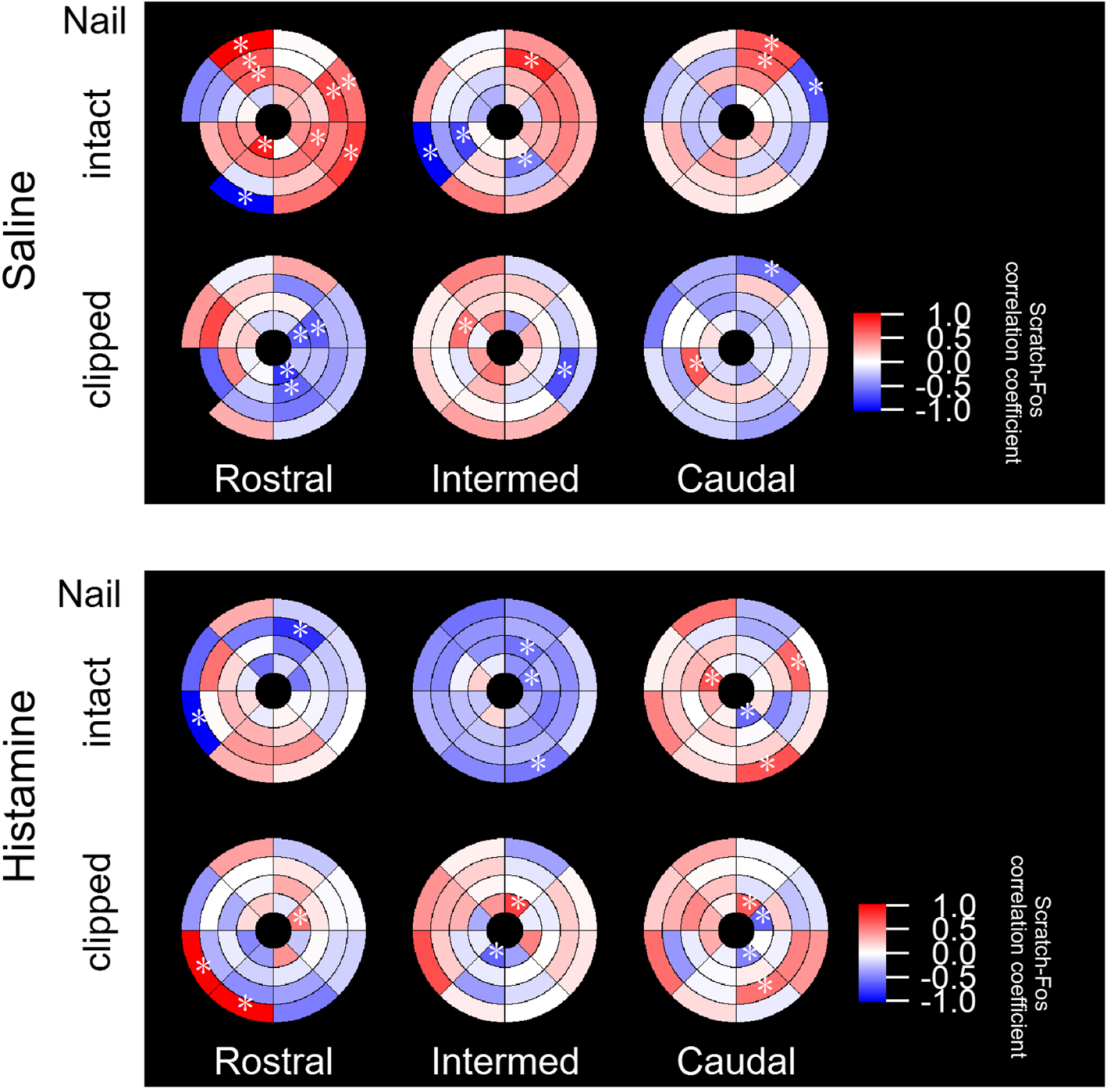
Scratch-Fos correlation coefficients in each eNAc region under four different itch/scratch conditions. Zone-and-sector plots for the Scratch-Fos correlation in saline- (upper panel) and histamine- (lower panel) injected mice. The upper and lower plots in each panel indicate the plots of nail-intact and nail-clipped mice, respectively. The colors in each region are Spearman's rank correlation coefficient between the fraction of Fos( +) cells in DAPI( +) ROIs (%) and the number of bouts of scratches observed in each mouse. Reddish and bluish colors indicate positive and negative correlations, respectively (values are shown in the color scale on the right). The regions marked with an asterisk indicate a significant correlation (The significance level is set at *P* < 0.1)

In the present study, we recorded scratching behaviors in all mice, in which Fos expression in the eNAc was analyzed. Therefore, we investigated whether the number of cells expressing Fos in specific regions correlated with the number of scratches in each mouse with different treatments. We estimated Spearman's non-parametric correlation between Fos expression in each region and the number of scratches recorded in a mouse for all regions (all correlograms used for correlation coefficient estimations are shown in Supplemental Fig. 6). We estimated Spearman's correlation coefficient between the fraction of Fos( +) in DAPI( +) cells and the total number of scratches (the "Scratch-Fos" correlation) for all regions and showed it on zone-and-sector plot heat maps with values ranging between -1 and 1 (Fig. 7). Regions exhibiting a positive Scratch-Fos correlation were shown in a reddish color and those with a negative correlation in a blueish color (Fig. 7). Saline-injected nail-intact mice exhibited more areas with a positive Scratch-Fos correlation, mainly in the rostral and intermed regions. In contrast, a negative correlation was observed in nail-clipped mice or there was no relationship. Nail-intact histamine-injected mice showed regions with a negative correlation mainly in the intermed plane, which was no longer detected in nail-clipped mice.

### The Scratch-Fos correlation was affected by distinct conditions and grouped into clusters distributed in distinct eNAc regions

**Fig. 8:**
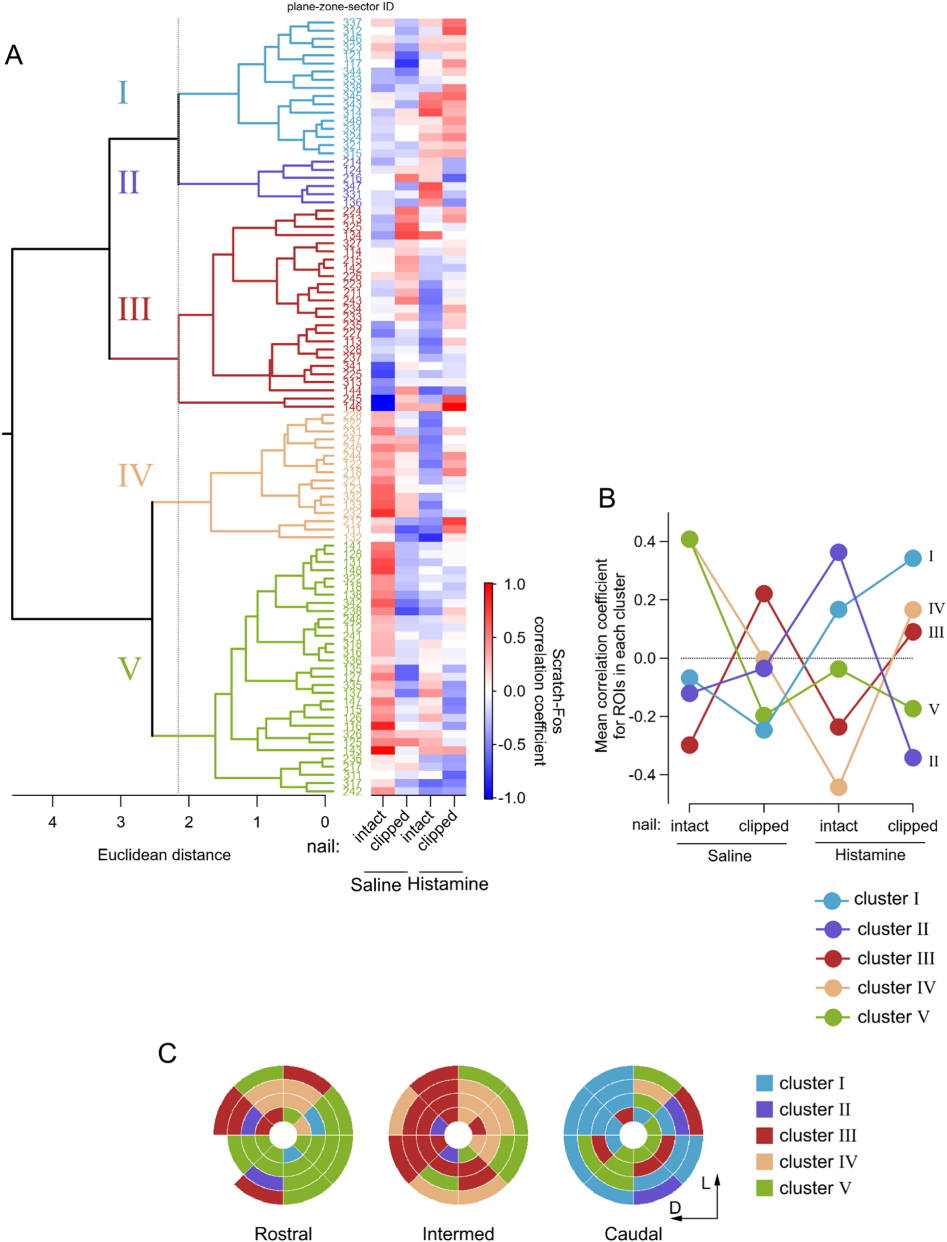
Hierarchical cluster analysis to categorize eNAc regions by Scratch-Fos correlations under different itch/scratch conditions. A hierarchical cluster analysis to categorize eNAc regions by scratching and Fos expression in four itch/scratch conditions. **A** Left: A dendrogram representing clusters based on the Scratch-Fos correlation coefficients of 95 regions (4 zones × 8 sectors × 3 planes, excluding zone 4-sector 5 in the rostral slice). The vertical dashed line indicates the Euclidean distance threshold used to separate the regions into 5 independent clusters. The three-digit numbers to the right of the dendrogram correspond to plane-zone-sector IDs (Table 1). Right: Heat map plot showing the Scratch-Fos correlation coefficient in each region under four itch/scratch conditions. The color is according to the scale on the right. **B** The mean Scratch-Fos correlation coefficient in each cluster under the different itch/scratch conditions. The numbers below the figure indicate the cluster numbers shown in Fig. 8A. **C** Zone-and-sector plots showing the clusters to which each region belongs. Cluster numbers are shown with a color index on the right

To reveal the spatial organization of regions with similar responses in the "Scratch-Fos" correlation among these regions, we conducted a hierarchical cluster analysis. The 95 regions (the correlation coefficient was not estimated in one of the 96 PZS regions because of an insufficient number of DAPI-positive ROIs) were classified into five clusters according to the similarity of correlation coefficients under the four conditions (Fig. 8A). The heatmap in Fig. 8A shows Scratch-Fos correlation coefficients under the four experimental conditions for each region. Figure 8B indicates the average correlation coefficients for each of the five clusters under the four conditions. The regions belonging to each cluster are shown in different colors in the zone-and-sector plots in Fig. 8C.

The regions in the ventromedial and dorsal lateral caudal eNAc (*cluster I*, Turkish blue in Fig. 8) were characterized by a high Scratch-Fos correlation only in mice unable to scratch sufficiently even if they wanted to (i.e., histamine-injected nail-clipped mice). In contrast, the ventromedial rostral eNAc (*cluster V*, matcha green in Fig. 8) showed the strongest correlation in housekeeping spontaneous scratching (i.e., saline-injected nail-intact mice). *Cluster IV* (light orange in Fig. 8), which was mostly located in the dorsal-rostral and dorsomedial-Intermed NAc, showed an interesting pattern in the correlation coefficient; the strongest correlation was observed in nail-intact saline-injected mice and the weakest in nail-intact histamine-injected mice, suggesting that increased activities in this cluster suppressed scratching in histamine-injected mice. In contrast, *cluster II* (purple in Fig. 8), which does not belong to the conventional map of the eNAc, showed the strongest Scratch-Fos correlation in nail-intact histamine-injected mice, suggesting that its activation is positively associated with scratching. The dorsolateral-intermed region (*cluster III*, red in Fig. 8) showed consistent patterns: under saline-injected and histamine-injected conditions, negative correlations were observed in nail-intact mice, while weak positive correlations were noted in nail-clipped mice. Therefore, there was anatomical consistency in how the correlation coefficient changed across the four modalities, revealing intriguing patterns within each cluster (Fig. 8C).

## Discussion

Here, we presented evidence that different subregions of the eNAc showed distinct activation patterns in response to itch/scratch behaviors with different modalities and conditions. This study is the first to propose that distinct subregions of the eNAc are separately activated in association with 1) the itch-induced urge to scratch, 2) scratching behaviors and their emotional outcome, and 3) spontaneous housekeeping scratching behavior without any pruritogenic stimulation. These novel results provide the following insights. The eNAc cannot be regarded as a single or simple assembly of a few anatomically defined units in complex emotion-accompanied and goal-oriented behaviors, such as the itch/scratch. Moreover, in future studies, distinguishing the rostral-caudal level of the eNAc will be very important for elucidating the anatomy-function relationship of the eNAc. In addition, different subregions of the eNAc form distinct groups of clusters, each with different implications in itch and its relief by scratching, as revealed by comparisons of neural activities (Fig. 6) and the scratch-activation correlation between distinct itch-scratch conditions (Fig. 8). Therefore, the unpleasantness of itchiness, the outcomes of scratching, such as its pleasantness, and the lowered affective efficacy of scratching are represented in distinct regions of the eNAc network. These points are discussed below.

### The Fos expression pattern did not match the conventional rough anatomical classification, but was organized into functionally relevant clusters

In the present study, we identified distinct clusters in the eNAc according to the expression of c-Fos under distinct itchiness conditions (with an intradermal injection of histamine) and scratching conditions (with clipped or intact nails) using an unbiased analysis of expression patterns. These results strongly suggest that neurons, depending on their three-dimensional localization, show functionally correlated responses to each distinct condition. These distinct but spatially segregated responses mainly result from similarities and differences in 1) the origins of projections that the neurons in each cluster receive, 2) the type of neuromediators used, and 3) the synaptic organization of intra-NAc connectivity [12, 14, 17, 22, 23]. The present results of spatially clustered activation patterns did not necessarily correspond with the conventional "core-and-shell" classification. This result would suggest that the expression of these three factors does not follow that simple concentric organization [12]. This conclusion is consistent with the proposal by Gangarossa et al., who detected five distinct "sub-territories" within the medial-to-ventral shell of the NAc using molecular expression and differences in extracellular signal-regulated kinase phosphorylation after dopamine-related drug administration [14]. A small sub-territory with the low expression of Drd2 (named the "D2R-expressing poor zone") in the medial shell, which was identified for the first time [14] was also clearly present in zone 4, sector 1 of the rostral and intermed planes in the present study (Fig. S5). These results support the idea that the NAc does not have a simple organization that allows for a simple classification, such as the "core and shell" of the subregions with D1R and D2R expression. For example, dopaminergic terminals from VTA neurons in the lateral shell, but not in the medial shell, are excited in synchrony with chloroquine- or endothelin-1-induced scratching [10] in a manner that is sensitive to D1R blockade in the lateral shell but not in the medial shell. In contrast, this scratching behavior was attenuated by the activation of D2R either in the lateral or medial shell [13]. Although these results suggest the strong involvement of scratching-induced dopamine release and the subsequent activation of D1Rs in the lateral shell, they also indicate other roles for D2Rs in both the lateral and medial shells in inhibiting scratching behaviors. Liang et al. (2022) also suggested that neurons with D2Rs in the medial shell were involved in the termination of scratching behaviors.

The medial-shell site of the D2R agonist microinjection in their study [13] overlapped with cluster β (Fig. 6B and C) and cluster IV (Fig. 8B and C) in the present study. As shown in the results obtained herein, the former was characterized by the highest Fos expression (Fig. 6B) and the latter by the lowest scratch-Fos correlation (Fig. 8B) in histamine-injected nail-intact mice. The increased expression of Fos in cluster β in Fig. 6 of histamine-injected mice was more potent in nail-intact mice than in nail-clipped mice, suggesting that high Fos expression in this cluster was related to the degree of affective outcomes of itch relief by scratch. This implies that vigorous scratching activates clusters β and IV, which may subsequently attenuate scratching behavior. Another cluster of interest is the cluster I (Fig. 8), which is characterized by a consistently strong (positive) scratch-Fos correlation, indicating that more scratching is associated with the higher expression of Fos in this cluster. Cluster β (Fig. 6) and cluster I (Fig. 8) did not overlap, suggesting that, possibly through an intermediary of intra-nucleus connections, these distinct clusters play opposite roles in the itch/scratch cycle, one as a brake and the other as an accelerator for scratching. A plausible concept based on these observations is that the balance in the interplay between distinct regions/clusters in the eNAc will affect the physiologically functional or aberrant and excessive itch-scratch relationship.

### What causes the regional difference in itch-associated activation in the eNAc?

The factors contributing to regional differences in histamine- and nail condition-dependent activation patterns in the eNAc warrant further study. In general, the shell and core are considered to present neurons with differing dendrite complexities, dopamine receptor expression, and different sources of glutamatergic and dopaminergic inputs [7, 14]. However, these differences are quantitative and neurons in the NAc, as well as the eNAc, share standard common features, such as medium spiny GABAergic neurons as part of the ventral striatum structure [14] and even of the extended amygdala [24]. In addition, our RNAscope analysis of the mRNA expression of Drd1 and Drd2 indicated that the shell and core do not simply depend on the expression of these receptors. Furthermore, we did not find significant differences between the composition of Drd1( +) and Drd2( +) neurons in the regions with increased and decreased Fos( +) expression by itch-scratch stimulations (Fig. 4A4 and B4 and Fig. 5A3 and B3), indicating that the expression pattern of dopamine receptors does not predict the responses of Fos expression.

#### Regional differences in external inputs to the eNAc

Another more plausible difference underlying distinct regional activation is specific inputs. Despite similarities in afferent and efferent projections of the core and shell, there are some notable differences that may account, in part, for their specialized behavioral functions. For example, the ventral subiculum of the hippocampus projects predominately to the shell, while the dorsal subiculum projects mainly to the core (reviewed in [25]). Projections to the NAc from the ventral subiculum play a stronger role in drug seeking and anxiety, while those from the dorsal hippocampus are more involved in memory and spatial navigation [26, 27]. In the rat, the medial prefrontal cortex is comprised of dorsal (prelimbic) and ventral (infralimbic) areas [28, 29] that selectively project to the NAc core and shell, respectively. The basolateral amygdala, known for its involvement in associative learning, sends projections to both the core and shell; however, these projections diverge from its anterior (core) and posterior (shell) parts, respectively [30–32]. Various brain-wide regions are activated in mice, revealed using c-Fos expression, and in human subjects, identified using functional MRI, under itch-inducing conditions [15, 33]. Since most of these regions showing itch-associated activation have connections to the NAc, it is possible that the differential activation of these areas may also underlie the region-specific activation patterns in the eNAc found in the present study.

It remains unidentified how the specific information on itch is sent to and activates NAc neurons. Primary afferent fibers activated by histamine and carrying pruriceptive information form synapses in the dorsal horn with neurons that mostly project to the parabrachial nucleus, which sends information to the central nucleus of the amygdala, the bed nucleus of the stria terminalis, the lateral hypothalamus, and other structures [34]. Further studies are needed to examine the pathways and mechanisms by which eNAc neurons receive this information, which may also provide insights into the regional differences observed in activation patterns in the present study.

#### Regional differences in outputs from the eNAc

In addition to distinct afferent inputs to the NAc core and shell, efferent projections also differ. For example, the NAc core predominantly sends projections to the motor regions important for executing subsequent actions, such as the ventral pallidum and subthalamic nucleus ([35, 36]. However, the shell predominantly sends projections to limbic areas, such as the lateral hypothalamus, bed nucleus of the stria terminalis, central nucleus of the amygdala, and ventromedial ventral pallidum [25, 35]. Therefore, the involvement of the core appears to be greater in goal-directed actions (e.g., necessary for learning about the environment), while that of the shell is greater in emotionally relevant information for encoding the value of stimuli in the environment. The Scratch-Fos correlation is interesting, but may reflect two different types of causalities: increased activity in regions driving scratching behavior and increased activity resulting from increased sensory inputs from other reward-associated structures, such as the VTA.

In addition to the differences observed in clusters on sector-and-zone dimensions, we revealed the involvement of distinct posterior-anterior planes of the eNAc in distinct itch/scratch behaviors, which has not yet been examined in detail. For example, the highest Scratch-Fos correlation in the rostral eNAc in nail-intact but not nail-clipped saline-injected mice was beyond our expectation (Fig. 7). This result suggests that these regions in the rostral eNAc with a significant Scratch-Fos correlation (Fig. 7; saline, nail-intact) play essential roles in the relationship between house-keeping scratching and its affective outcomes, which is perturbed by a pruritogen injection.

### What are the effects of nail clipping?

A remaining question is the mechanisms underlying the effects of nail clipping. The fraction of Fos( +) cells in the eNAc decreased in most regions regardless of the saline or histamine injection (Fig. 5A1, A2, B1 and B2). The simplest interpretation of this effect is that reduced sensory inputs resulting from scratching without nails decreased the number of Fos(+) NAc neurons in most regions. However, three observations argue against these simple interpretations. In some regions, the effects of nail clipping were stronger in saline-injected mice than in histamine-injected mice (Fig. 5A2 and B2), indicating that c-Fos expression does not simply depend on somatosensory inputs. Furthermore, the effects of histamine on Fos expression depended not only quantitatively, but also qualitatively on the nail status, as shown with the histamine-induced decrease in Fos( +) in a subset of regions in nail-intact mice (Fig. 4A3), which was absent in nail-clipped mice (Fig. 4B3). In addition, the Scratch-Fos correlation markedly differed between saline- and histamine-injected mice, particularly in nail-intact mice (Fig. 7), indicating that the effects of sensory inputs on scratching were not a linear consequence. A more plausible interpretation is that some subregions in the eNAc positively or negatively represented differences in or a discrepancy between the motor command to scratch and its expected outcome (i.e., subsequent pleasantness, which may be represented by the strength of dopaminergic inputs [9]). Histamine may increase the urge to scratch, subsequent unpleasantness, and the expectation of the itch to be relieved by scratching. These may have been perturbed by nail clipping. Therefore, specific subregions may represent these different aspects of the motivational link between the unpleasantness of itch, the urge to scratch, and subsequent outcomes, such as satisfaction [3]. Another possible interpretation for the significant increase in the scratch number in nail-clipped mice is that scratching without nails was more pleasurable because it was devoid of nociceptive components resulting from skin damage caused by nail scratches. If this is the case, the interpretations described above based on the lowered scratch outcomes with nail-less scratching need to be reconsidered. However, the apparent absence of changes and differences in skin condition after the observation period between the four groups argues against this interpretation. An analysis of the activation time course of eNAc activity will provide insights into this possibility, as discussed in the next section. Lastly, an unexpected but intriguing observation was the significant difference in Fos expression in some regions between nail-intact and nail-clipped mice, even in the saline-injected group (Fig. 5A2). This result implies that the perturbed scratch efficacy by nail clipping in spontaneous housekeeping scratch affects neuronal excitation in specific areas in the eNAc. The central mechanisms underlying the spontaneous scratch behavior or active touch on the body might differ from those playing roles in evoked scratch responses with their different physiological functions [37, 38].

### A limitation of the present study

In this study, we used the term "activated" for regions with a higher probability of c-fos mRNA expression. Differences in the degree of Fos expression between regions may represent different aspects of eNAc activity, including 1) increased firing causing large Ca^2+^ entry, 2) increased excitatory synaptic inputs activating Ca^2+^-permeable receptor channels, such as NMDA receptors, and 3) increased excitatory inputs activating metabotropic glutamate receptors. In addition, the 45-min time period from the histamine injection to brain sampling may reflect the expression of fos mRNA with different time courses. The number of scratches peaked approximately 15 min after the histamine injection but persisted until 45 min, particularly in the nail-clipped mice (Fig. 1A). A potential consequence of different time courses is a stronger urge to scratch in the early period followed by higher affective effects of scratching in nail-intact mice or lower scratch efficacy, particularly in the later period in nail-clipped mice. Developing techniques that enable observations with a higher time resolution while maintaining high spatial resolution will provide insights into the spatial and temporal dynamics of eNAc neuron activities.

### Future perspective

The present unbiased model-free analysis revealed spatially distinct activation patterns in the so-called "reward center" in the different modalities of itch-scratch behaviors for the first time. In addition, these approaches clarified the differential roles of dopaminergic signaling in different aspects of the itch-scratch cycle. A more detailed analysis of the network organization within the eNAc with respect to differential synaptic inputs from dopaminergic neurons in the VTA and glutamatergic neurons in the other structures, as well as intra-nucleus synaptic connectivity underlying responses to sensory inputs and the promotion of stereotypic motor behaviors, will advance our understanding of the relationship between the itch-induced urge to scratch and scratch-induced relief.

A reference table for the simplified representation of plane-zone-sector IDs. In this simplified form, the location of each plane, zone, and sector is shown with three-digit numbers. For example, a PZS ID of "212" refers to Fos expression in the region in plane = intermed, zone = 1 and sector = 2. This simplified form is used in Figs. 6 and 8 and in S6 for the visibility of figures.

## Methods

The manipulation of animals was approved by the Institutional Animal Care and Use Committee (IACUC) of The Jikei University School of Medicine (Approval No. 2015–019, 2017–049, and 2022–014) and conformed to the Guidelines for Proper Conduct of Animal Experiments of the Science Council of Japan (2006) and the guidelines of the International Association for the Study of Pain [39].

### Animals

Male adult C57BL/6 J mice (age; 7–8 weeks old) were obtained from Charles River Laboratories Japan, Inc. (Yokohama, Japan) and were housed on a 12-h light/dark cycle with free access to food and water.

### Induction and evaluation of scratching behavior

All mice used in this study underwent 1) implantation of a small magnet ring in the bilateral hindlimb, 2) bilateral hindpaw nail-clipping (the control was nail-intact), and 3) intradermal injection of histamine (the control was saline). The details of these operations are described below.

#### Implantation of a small magnet

Seven days before the day of scratching recordings (Day 1 in Fig. S1), all mice were anesthetized with isoflurane to shave their nape, insert subcutaneous magnets into the bilateral hind paws, and clip the nails on the bilateral hind paws. Briefly, mice had the skin on the dorsum of the hind paws cut with scissors, a small magnet (1 mm in diameter, 3 mm in length) was inserted with tweezers, and the wound was closed with a bond. This operation did not affect the general behavior of mice.

#### Nail clipping

Mice were randomly divided into two groups: those with and without clipped nails on the bilateral hindlimbs ("nail-clipped" and "nail-intact", respectively) (Fig. S1, Day1). The nails of all digits on bilateral hind paws were clipped using scissors for mice assigned to the "nail-clipped" group. Nails remained intact in the "nail-intact" group. Mice recovered from anesthesia and were placed into the home cage.

#### Handling and Habituation

Two days later, mice were handled for habituation to the experimenter for five days (Fig. S1, Day3). Mice were then habituated to the barrel-shaped recording chamber (10.8 cm in diameter, 18 cm in height) used to count the number of scratches for three days. An injection needle was touched against the shaved skin area several times for three days for habituation (Fig. S1, Day5).

#### Histamine injection and scratching behavior recording

After repeated handling and habituation to the experimenter and recording chamber for one week, mice were further divided randomly into two groups: those for testing the effects of an intradermal injection of histamine and those of saline ("histamine" and "saline", respectively) (Fig. S1, Day 8).

On the day of scratching recording, mice were habituated to the recording chamber for at least 15 min. Mice were removed from the recording chamber and intradermally injected at the nape with histamine (200 μg/20 μL saline; #H7250, Sigma-Aldrich, Tokyo, Japan) or saline (20 μL). Mice were returned to the recording chamber immediately and scratching behavior was recorded for 45 min (Fig. 1A). Scratching behavior was detected using MicroAct® (Neuroscience, Tokyo, Japan). This system is based on the detection of an electric current in the coils according to the movement of the magnets implanted in the hind paws and the measurement of the occurrence time and duration of each scratch bout. After sampling the brain, it was confirmed that magnets remained in both hind paws in all mice (Fig. S1).

### The mice cohort size

As described above, all mice used in this study underwent 1) histamine or saline injection and 2) nail-clipping or nail-intact, resulting in four cohorts of mice (Fig. S1). The numbers of mice in each cohort were *n* = 12 for the histamine-injected + nail-intact group, *n =*12 for saline-injected + nail-intact group, *n =*13 for histamine-injected + nail-clipped group, and *n =*11 for saline-injected + nail-clipped group.

### Tissue preparation

Mice were decapitated under 5% isoflurane, and the brain block containing the NAc was dissected and frozen within 5 min. A coronal brain block was freshly frozen using isopentane chilled with dry ice, and stored at -80 °C for up to 2 days. Brain blocks were embedded in OCT compound, and a series of 16-µm-thick coronal sections containing the NAc were prepared with a cryostat (CM1850, Leica Biosystems, Tokyo, Japan). The sections collected were equivalent to the rostrocaudal level from bregma 1.70 mm to 0.86 mm in the Mouse Brain Atlas [40]. Brain sections were stored at − 80 °C for up to 2 days.

### Multiplex FISH

FISH was performed using Multiplex fluorescent RNAscope (Advanced Cell Diagnosis [ACD], Hayward, CA, USA; Medical & Biological Laboratories, Tokyo, Japan). The following probes were obtained: Drd1 (Mm-Drd1a-C2, #406,491-C2), Drd2 (Mm-Drd2-C3, #406,501-C3), and Fos (Mm-Fos, #316,921). The RNAscope protocol was followed as indicated in the user manual with adjustments. Briefly, after fixation in 10% neutral-buffered formalin at 4 °C for 15 min, sections were washed with PBS. Sections were then dehydrated in four 5-min dehydration steps in 50, 70, 100, and 100% ethanol. Sections were incubated with protease III (diluted 1:1 with PBS) for 30 min. Fos, Drd1, and Drd2 probes were mixed 50:1:1 and pipetted onto each slide. Probe hybridization and amplification steps were performed according to the standard instructions of the manufacturer. Sections were incubated with DAPI for approximately 30 s, mounted with Aqua Poly/Mount (Polysciences, Warrington, PA) on coverslips, and then stored at 4 °C.

### Image acquisition and multiple image alignment (MIA)

All fluorescence images were obtained using the laser-scanning confocal microscope, FV1200 Confocal microscope equipped with 405, 473, 559, and 635 nm laser lines and high-sensitivity GaAsP detectors, with the filter cube FV12-MHYR, using an UPlanSApo 20 × /0.75 NA objective (Olympus, Japan). Multiple overlapping adjacent images of the NAc were taken on a moving stage manually and saved as OIB files (1024 × 1024 pixels). Images of the right side of the brain were captured in this study. A series of OIB files from one brain section were aligned using the MIA function of CellSens imaging software (Olympus, Japan). Multiple images were "stitched" with individual neighboring images into a large "stitched" image without resolution loss by the maximum correlation-based optimization of the software. MIA was performed based on the DAPI channel image (Overlap mode; linear). Each four-channel stitched image was saved as a 16-bit tiff file for subsequent image analysis (Fig. S2A). We positioned images such that the eNAc was dorsal side up and the most medial side was to the right of each image.

### ROI definition with the ROI manager in ImageJ

Image data analyses were performed in two steps. The saved tiff file was read by ImageJ/Fiji software (NIH image) for the primary detection of ROIs using the ROI manager. Each of the four channels corresponding to the signals for DAPI, Drd1, Drd2, and Fos in the 16-bit tiff format was read into ImageJ. To detect and register cells, we used DAPI signals by thresholding the DAPI channel image with the Huang method and binarizing using "watershed" separation to prevent detected cells from sticking together (steps 3 and 4 in Fig. S2B). Areas with DAPI signals, likely to correspond to each cell, were numbered and defined as ROIs. The "Analyze Particles" plug-in was applied to these ROIs to measure their characteristics (location, size, and shape properties). We then binarized the signals for the Drd1, Drd2, and Fos channels using Reny entropy methods (step 8 in Fig. S2B) and evaluated the area overlap with each ROI identified according to the DAPI signal using the ROI manager (step 10 in Fig. S2B). At this point, we did not select the detected areas according to the size and circularity of ROIs, which was performed after plotting these features (Fig. S3D). The measured values for Drd1, Drd2, and Fos in individual ROIs were saved in a comma-separated value format together with an 8-bit composite tiff image for further analyses, as described below (step 11 in Fig. S2B).

### Expression analysis based on defined ROIs and visualization using a homemade code—"RNAscopeProcessor" on Igor 9

Using the values for each ROI obtained by ROI manager and the original Tiff image, fluorescence signals with each coordinate in slices were identified and analyzed using a homemade code for Igor 9.0 (Wavemetrics).

A composite 8-bit- tiff image of RNAscope that was stitched to cover the accumbens and surrounding structures was loaded together with the results of the ROI manager for each of the three channels (Drd1, Drd2, and Fos). Only apparent artifacts (debris on the slice and tissue overlap) in the image were carefully removed by the drawing tool (Fig. S3A). The detection quality of ROIs was visually confirmed using a zoom-up window showing the tiff image at a higher magnitude with circles showing the diameters of ROIs detected by the particle analysis of ImageJ (white circles drawn on the three-channel image in Fig. S3A and C) and thresholds for the diameter and circularity were fixed with cursors (Fig. S3C and D).

### Definitions of zones 1–4, sectors 1–8, and rostro-caudal *planes*

We defined the following three dimensions to describe the regions of the NAc and surrounding regions (hereinafter called "eNAc"): "zones", "sectors", and "planes".

Defining zones. Concentric circles defining the boundaries between zones were drawn as follows: 1) Circle 1 was drawn around the aco for the boundary between the aco and zone 1. This border was clearly defined because there were almost no neurons within this border, which made it likely to be an outer edge of the region mostly containing rostrocaudally passing axons. 2) Circle 4 was then drawn so that the dorso-medial part of the circle overlapped with the boundary set at the outermost expression of Drd1 and Drd2, forming a border between the NAc and lateral septum nucleus. 3) Two circles (2 and 3) were then positioned between circles 1 and 4 using the observed differences in cell density, which were noted as faint gaps between regions. The radii of these circles are summarized in Table 2. The regions bound by each circle were named Zone 0 (zone within the aco), and Zone 1 (the zone between circle 1 and circle 2), Zone 2, Zone 3, Zone 4, and Zone 5 were then defined accordingly.

**Table 2:** Summary of radii of five circles used for zone definition

	Rostral (*n =*41)	Intermed (*n =*45)	Caudal (*n =*42)
Circle 1 (between the aco and zone 1)	280 ± 24 (250—300)	293 ± 17 (250—300)	287 ± 22 (250—300)
Circle 2 (between zones 1 and 2)	591 ± 52 (500—730)	618 ± 62 (500—800)	570 ± 46 (470—670)
Circle 3 (between zones 2 and 3)	887 ± 72 (800—1060)	915 ± 69 (800—1170)	836 ± 71 (670—950)
Circle 4 (between zones 3 and 4)	1310 ± 135 (1010—1750)	1324 ± 134 (1060—1600)	1147 ± 152 (850—1460)
Circle 5 (between zones 4 and 5)	1662 ± 140 (1350—2030)	1732 ± 78 (1550—1900)	1648 ± 129 (1340—1900)

Values are shown as pixel numbers (1 pixel = 0.585 µm). Mean ± S.D. (range in parentheses)

#### Defining sectors

After defining the zones in each plane, eight "sectors" each with a 45° angle, starting from the sector immediately above the horizontal line and most medial (sector 1) and numbered counter-clockwise to sector 8 (Fig. 3), were made.

#### *Defining planes*

Three anterior–posterior sections from the rostral, intermed (abbreviated as "intermed"), and caudal NAc were defined (Fig. S3) based on the atlases by Franklin and Paxinos [40] and the Allen Institute [41]. The rostral plane represented (relative to the bregma) 1.78, 1.70, and 1.54 mm coronal sections, the intermed plane, 1.42, 1.34, and 1.18 mm sections, and the caudal plane, 1.10, 0.98, and 0.86 mm sections (Fig. S3E).

With these three dimensions, we classified the eNAc into 96 regions (4 zones × 8 sectors × 3 planes) and performed unbiased analyses of mRNA expression (Fig. S3F). In the Results section, regions were presented with three zone-and-sector plots each for one plane.

Using the results of RNAscope imaging, cells expressing Drd1, Fos, and Drd2 were identified, and thresholds for each signal were selected based on the percentage overlap with the DAPI area. Specifically, Drd1 was defined as positive when overlapping with DAPI by 15% or more, Fos by 20% or more, and Drd2 by 30% or more (Fig. S4A, B, C). The number of cells identified as "positive" for Fos, Drd1, or Drd2 was presented as the fraction of the total number of cells identified with a DAPI signal in each of the 96 regions defined above.

### Statistical analysis

Statistical analyses were performed using EZR [42] and Igor 9.0. The total number of scratches were compared using a two-way ANOVA in each timepoint (Fig. 1A) and one-way ANOVA followed by a post hoc analysis with Bonferroni corrections (Figs. 1B). The distribution of the scratch duration in each timepoint was compared using the Mann–Whitney-Wilcoxon test (Fig. 1C). A probability (P) smaller than 0.05 was considered to be significant (Fig. 1A, B, and C). In multiple comparisons of eNAc subregions, *P* values were corrected using the false discovery rate correction (Benjamini & Hochberg), and corrected values are shown as Q values (Figs. 4A3, 4B3, 5A2, and 5B2). In these cases, t values were presented only for regions with a significance level of Q < 0.1 after false discovery rate corrections (Benjamini & Hochberg). To compare the expression of dopamine receptors, the Kruskal–Wallis test, followed by a post hoc analysis using the Bonferroni correction or the Mann–Whitney U test, was performed (Figs. 4A4, B4, 5A3, and B3). Comparisons of the percentage of dopamine receptors co-expressed with Fos( +) cells were conducted using the chi-squared test (Figs. 4A5, B5, 5A4, and B4). A hierarchical cluster analysis was performed using the values of Fos( +) fractions (Fig. 6) and the Scratch-Fos correlation (Fig. 8) using the hierarchical cluster analysis implemented in Igor 9. We used the Euclidean dissimilarity metric with a Minkowski *P* value of two. The dendrogram was made with the complete linking method. Spearman's rank correlation coefficients were used in the Scratch-Fos correlation, and *P*-values < 0.1 were considered to be significant (Fig. 7). Graphs were generated using Igor 9.0 with codes written by one of the authors (F.K.). Analyses were performed by S.I.S. and F.K. in a blinded manner without knowledge of the mouse groups.

### Supplementary Information

Additional file 1: Figure S1. Experimental design. Figure S2. Setting of ROIs and their application to RNAscope signals of each channel. Figure S3. Definition of the eNAc on images and the selection of valid ROIs. Figure S4. Detection of cells expressing the mRNA of each molecule in the eNAc. Figure S5. Expression of dopamine receptors in the eNAc of saline-injected, nail-intact mice. Figure S6. Scattered correlograms for the Scratch-Fos expression relationship in all regions.

## Data Availability

The datasets and analysis program (Igor 9 code) used in the present study are available from the corresponding author upon reasonable request.

## Abbreviations

NAc: The nucleus accumbens
Drd1: Gene for the dopamine D1 receptor (D1R)
Drd2: Gene for the dopamine D2 receptor (D2R)
VTA: The ventral tegmental area
eNAc: Extended NAc
aco: The anterior commissure, olfactory limb
intermed: Intermediate
DAPI: 4',6-Diamidino-2-phenylindole
ROI: Region of interest
PZS: IDs the plane-zone-sector IDs
FISH: Fluorescence in situ hybridization
MIA: Multiple image alignment
