# Supplementary material for: Region-specific activation in the accumbens nucleus by itch with modified scratch efficacy in mice – a model-free multivariate analysis
Source: Mol Brain. 2024 May 23;17:27. doi: 10.1186/s13041-024-01101-w (PMC11119306; doi:10.1186/s13041-024-01101-w)
Supplement: Supplementary file 1 — Additional file 1: Figure S1. Experimental design. Figure S2. Setting of ROIs and their application to RNAscope signals of each channel. Figure S3. Definition of the eNAc on images and the selection of valid ROIs. Figure S4. Detection of cells expressing the mRNA of each molecule in the eNAc. Figure S5. Expression of dopamine receptors in the eNAc of saline-injected, nail-intact mice. Figure S6. Scattered correlograms for the Scratch-Fos expression relationship in all regions. [file 13041_2024_1101_MOESM1_ESM.pdf]

Day 1

- Magnet implantation to the left hindlimb
- Hindlimb nail clipping on half of the mice

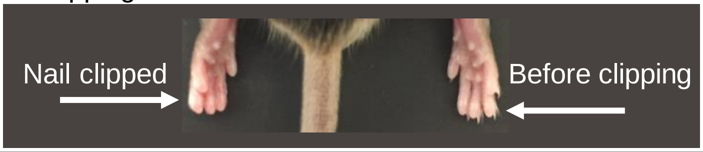

Day 3

- Handling (Day 3-Day 7, once a day)

Day 5

- Habituation to the recording chamber (Day 5 - Day 7, once a day)
- Habituation to the injection needle (Day 5 - Day 7, once a day)

Day 8

- Drug injection and scratch behavior recording
  1. Habituation in the MicroAct® (15 min)
  2. Histamine or saline i.d. injection at nape of the neck

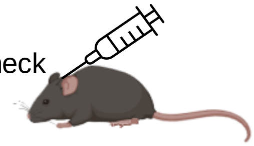

Numbers of mice		i.d. injection	
		histamine	saline
bilateral hindlimb	nail-clipped	13	11
	nail-intact	12	12

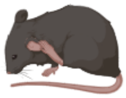

  3. Scratching recording using MicroAct® (45 min)
  4. Brain sampling

In situ hybridization

- Cryostat sectioning of the brain containing the N. accumbens
- Multiplex fluorescent in situ hybridization with RNAscope® (Fos, Drd1, Drd2) + DAPI staining

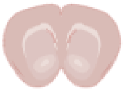

New Fig. S1

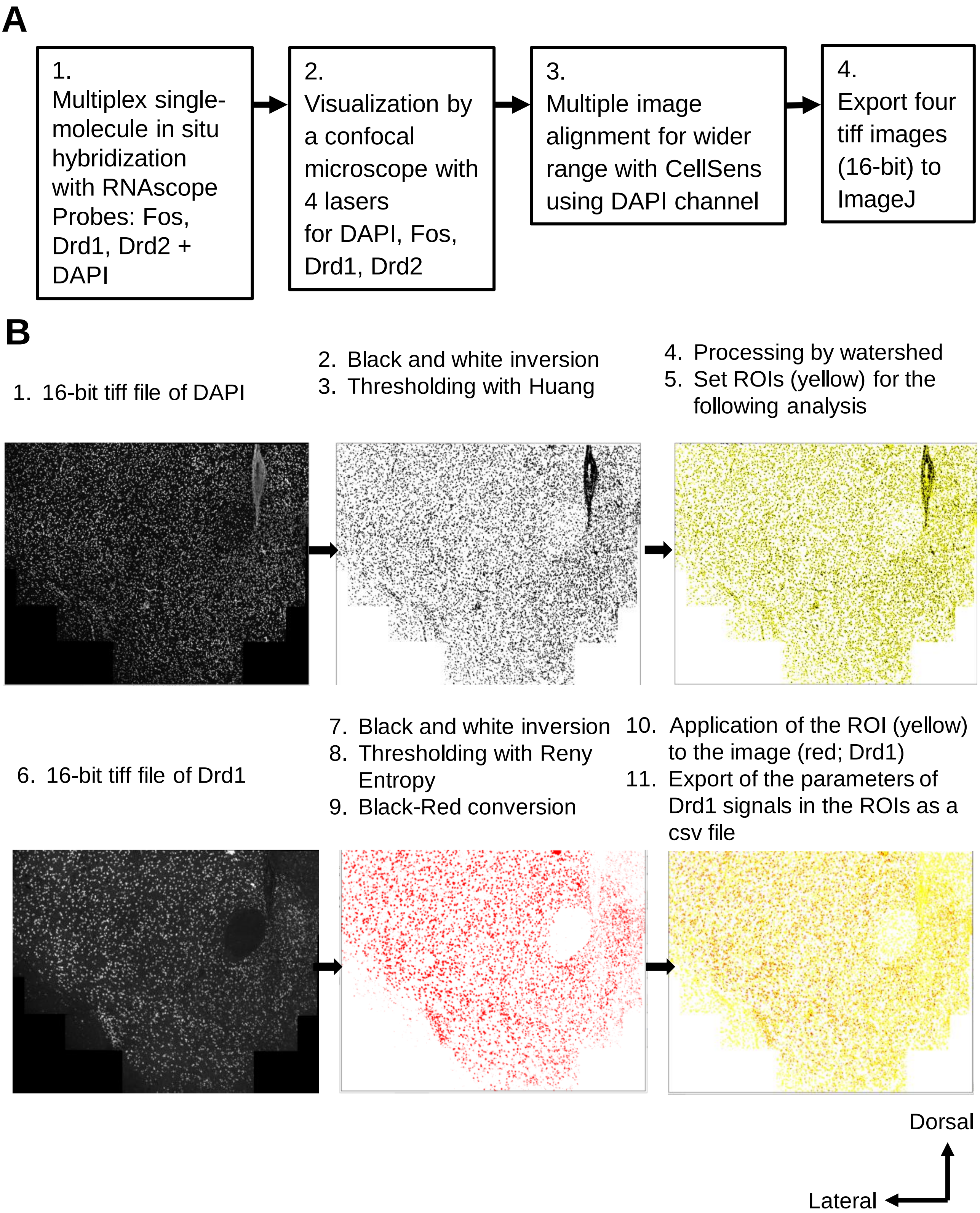
A
1. Multiplex single-molecule in situ hybridization with RNAscope Probes: Fos, Drd1, Drd2 + DAPI
2. Visualization by a confocal microscope with 4 lasers for DAPI, Fos, Drd1, Drd2
3. Multiple image alignment for wider range with CellSens using DAPI channel
4. Export four tiff images (16-bit) to ImageJ
B
1. 16-bit tiff file of DAPI
2. Black and white inversion
3. Thresholding with Huang
4. Processing by watershed
5. Set ROIs (yellow) for the following analysis
6. 16-bit tiff file of Drd1
7. Black and white inversion
8. Thresholding with Reny Entropy
9. Black-Red conversion
10. Application of the ROI (yellow) to the image (red; Drd1)
11. Export of the parameters of Drd1 signals in the ROIs as a csv file
Dorsal
Lateral

New Fig. S2

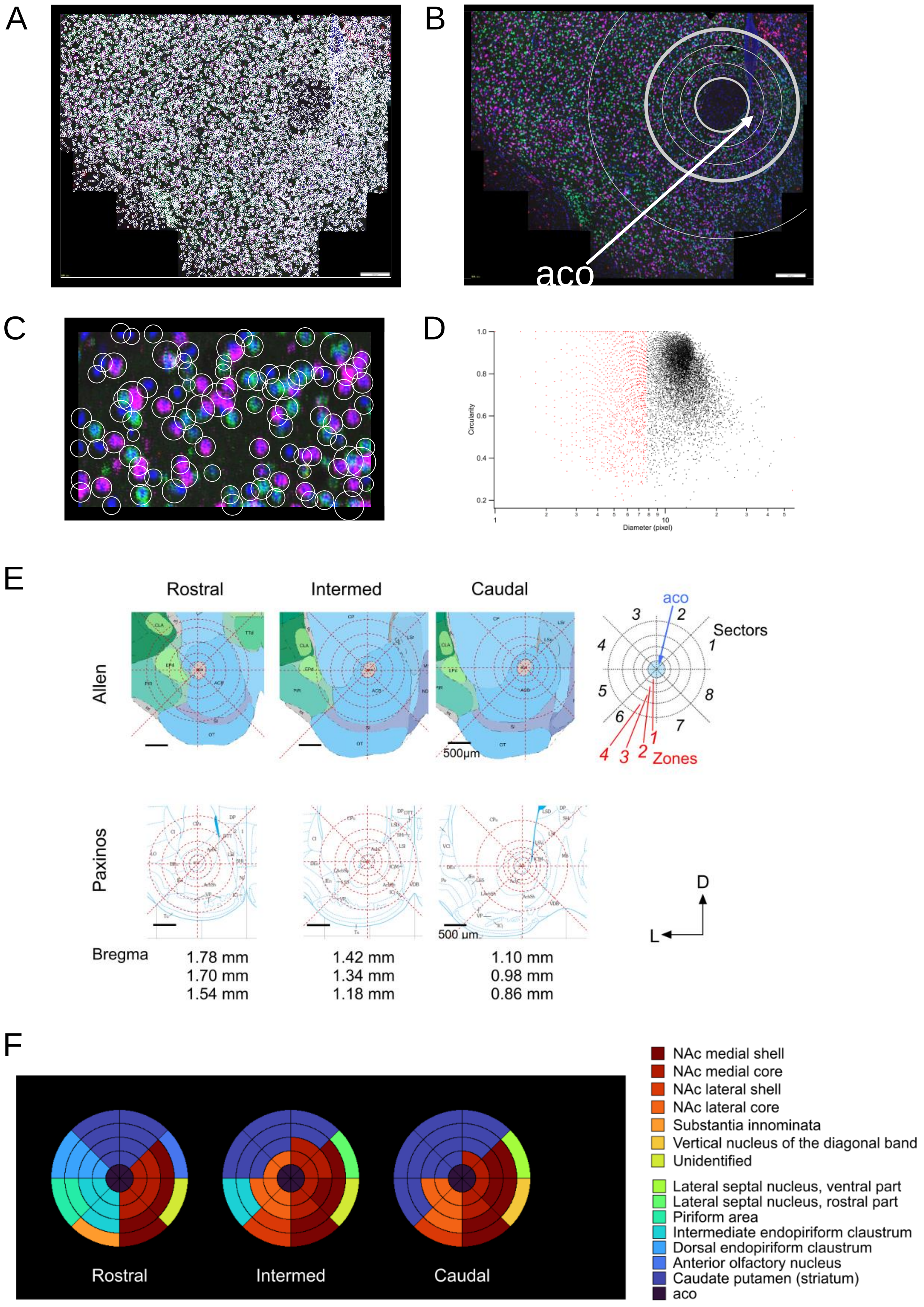
A
B
aco
C
D
Circularity
Diameter (pixel)
E
Rostral
Intermed
Caudal
Allen
500µm
aco
Sectors
Zones
Paxinos
500 µm
D
L
Bregma
1.78 mm
1.70 mm
1.54 mm
1.42 mm
1.34 mm
1.18 mm
1.10 mm
0.98 mm
0.86 mm
F
Rostral
Intermed
Caudal
NAc medial shell
NAc medial core
NAc lateral shell
NAc lateral core
Substantia innominata
Vertical nucleus of the diagonal band
Unidentified
Lateral septal nucleus, ventral part
Lateral septal nucleus, rostral part
Piriform area
Intermediate endopiriform claustrum
Dorsal endopiriform claustrum
Anterior olfactory nucleus
Caudate putamen (striatum)
aco

New Fig. S3

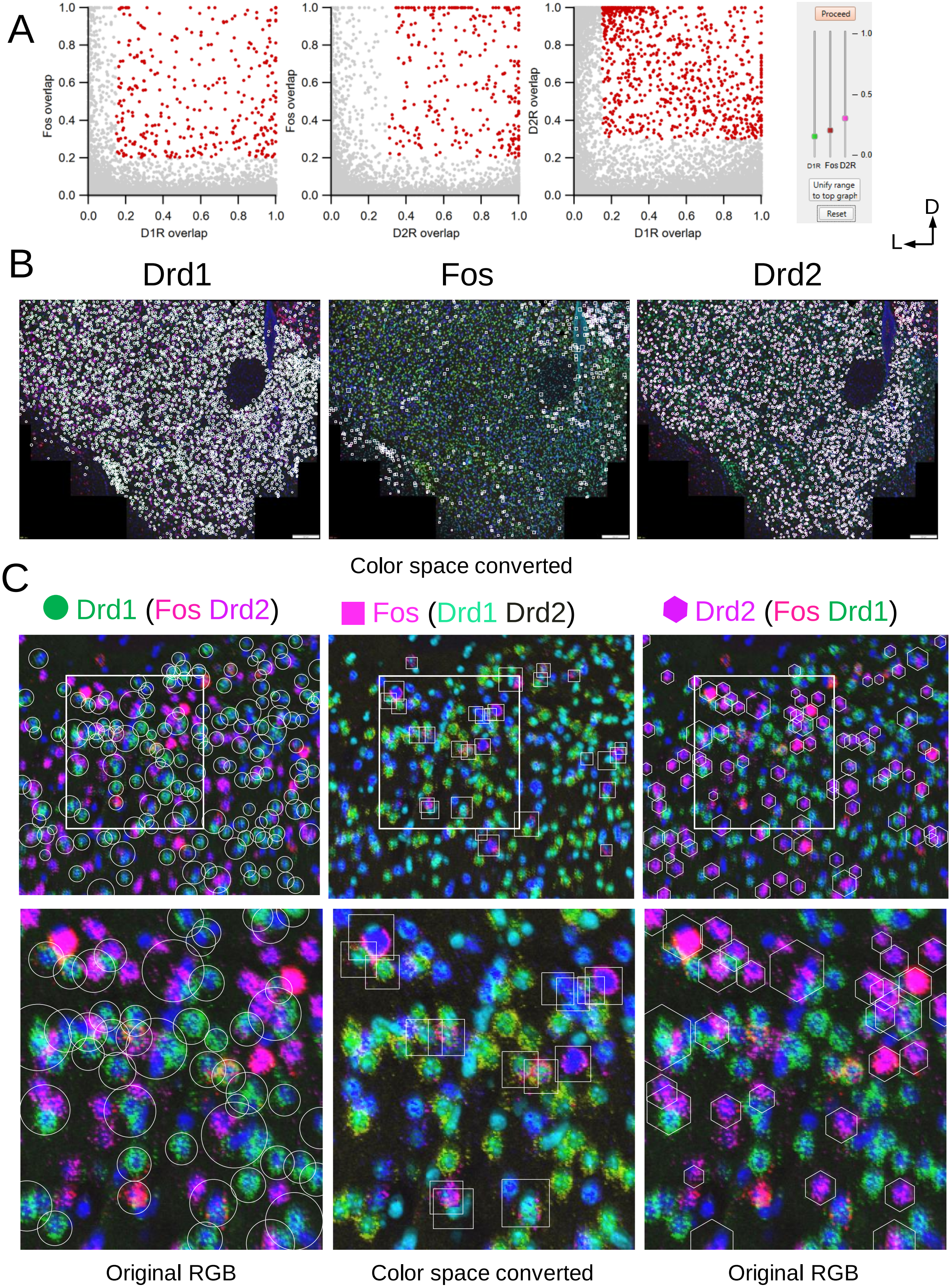
A
Fos overlap
D1R overlap
D2R overlap
D2R overlap
D1R overlap
Proceed
D1R Fos D2R
Unify range to top graph
Reset
D
L
B
Drd1
Fos
Drd2
Color space converted
C
Drd1 (Fos Drd2)
Fos (Drd1 Drd2)
Drd2 (Fos Drd1)
Original RGB
Color space converted
Original RGB

New Fig. S4

A

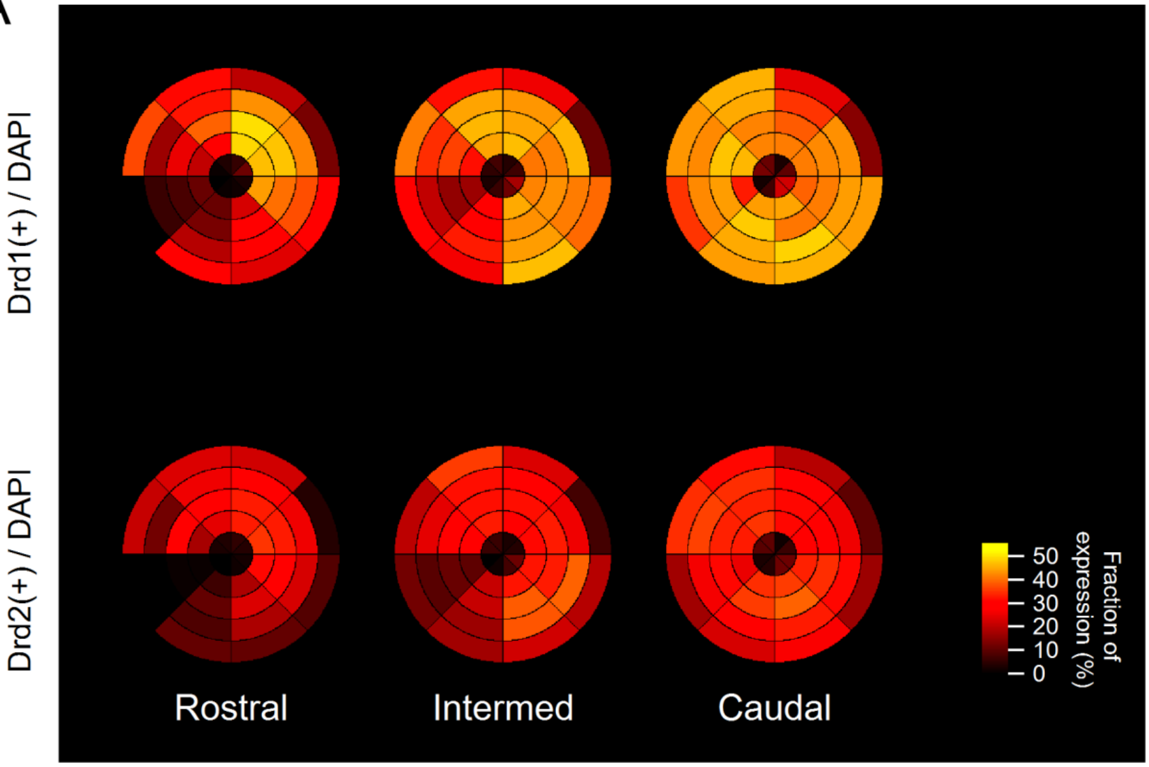
Drd1(+) / DAPI
Drd2(+) / DAPI
Fraction of expression (%)
50
40
30
20
10
0
Rostral
Intermed
Caudal

B

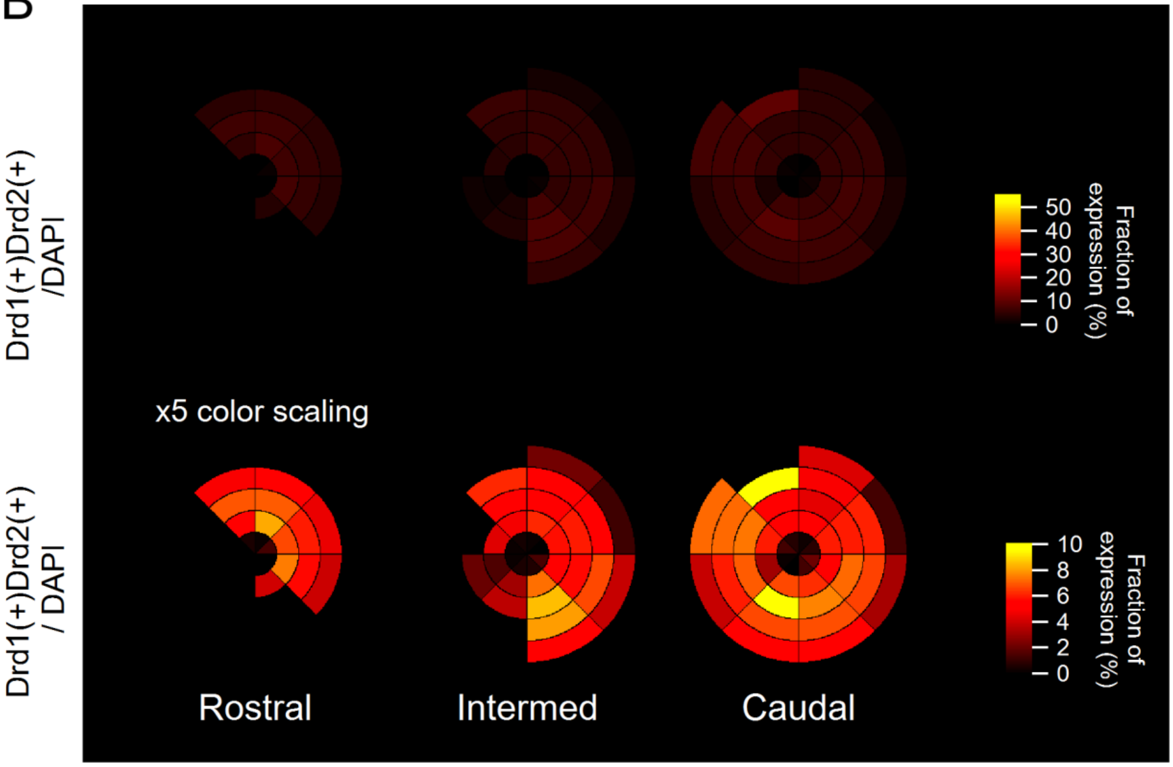
Drd1(+)Drd2(+) /DAPI
Fraction of expression (%)
50
40
30
20
10
0
x5 color scaling
Drd1(+)Drd2(+) / DAPI
Fraction of expression (%)
10
8
6
4
2
0
Rostral
Intermed
Caudal

New Fig. S5

A

Saline-injected nail-intact

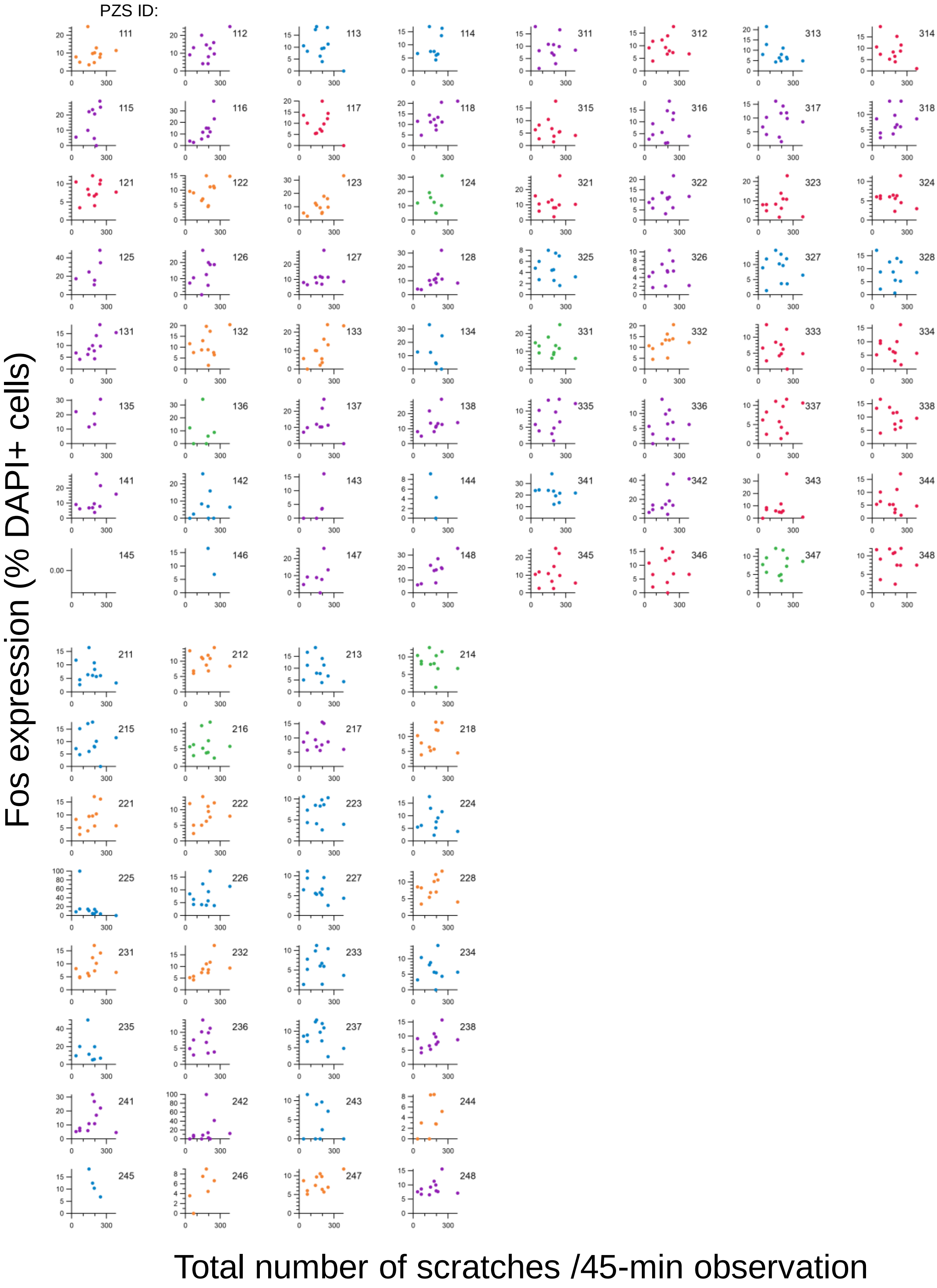

New Fig. S6

B

Saline-injected nail-clipped

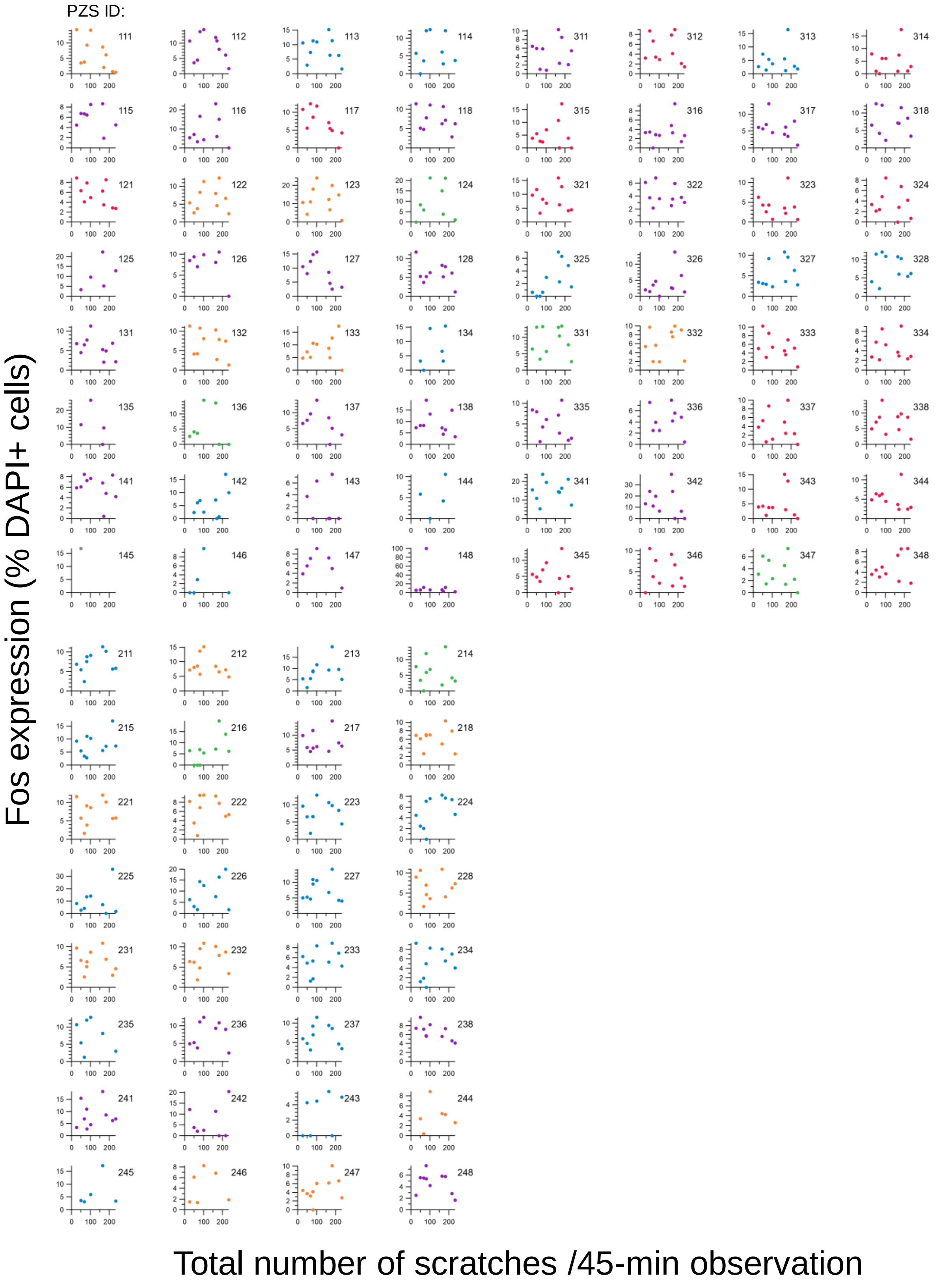

New Fig. S6

C

Histamine-injected nail-intact

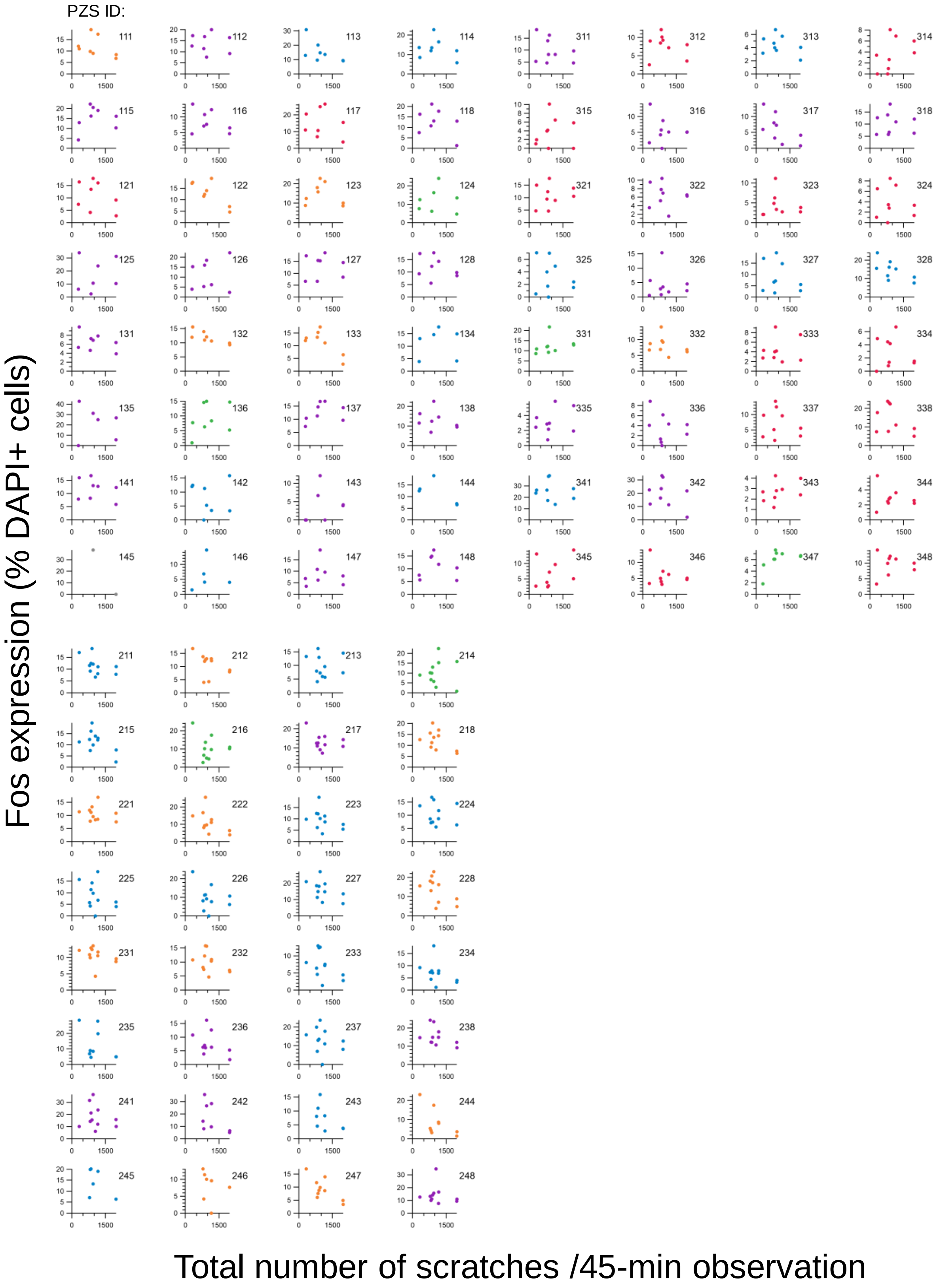

New Fig. S6

D

Histamine-injected nail-clipped

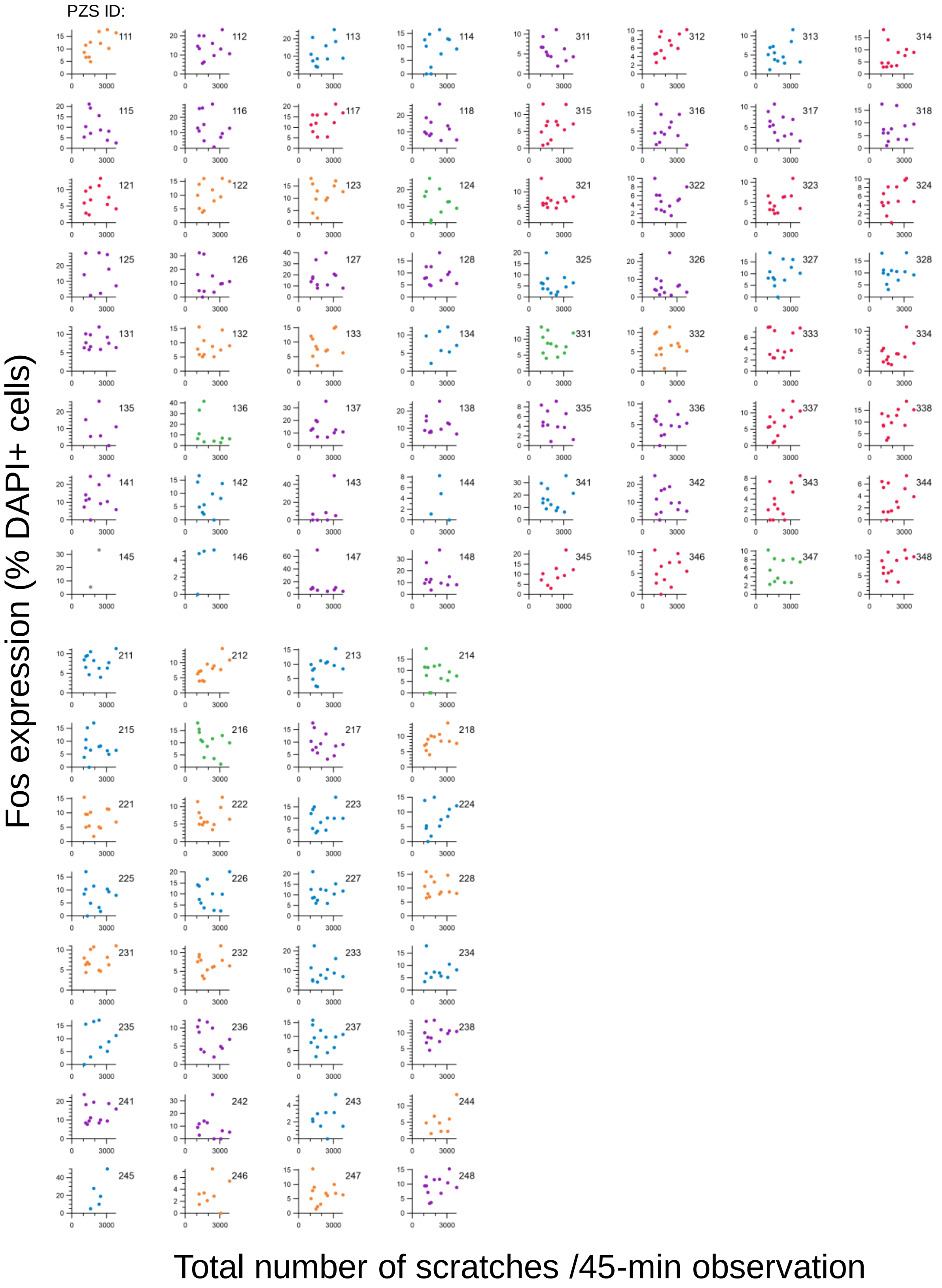

New Fig. S6
